# Who Is at Risk of Poor Mental Health Following Coronavirus Disease-19 Outpatient Management?

**DOI:** 10.3389/fmed.2022.792881

**Published:** 2022-03-14

**Authors:** Katharina Hüfner, Piotr Tymoszuk, Dietmar Ausserhofer, Sabina Sahanic, Alex Pizzini, Verena Rass, Matyas Galffy, Anna Böhm, Katharina Kurz, Thomas Sonnweber, Ivan Tancevski, Stefan Kiechl, Andreas Huber, Barbara Plagg, Christian J. Wiedermann, Rosa Bellmann-Weiler, Herbert Bachler, Günter Weiss, Giuliano Piccoliori, Raimund Helbok, Judith Loeffler-Ragg, Barbara Sperner-Unterweger

**Affiliations:** ^1^Department of Psychiatry, Psychotherapy, Psychosomatics and Medical Psychology, University Hospital for Psychiatry II, Medical University of Innsbruck, Innsbruck, Austria; ^2^Data Analytics as a Service Tirol, Innsbruck, Austria; ^3^Department of Internal Medicine II, Medical University of Innsbruck, Innsbruck, Austria; ^4^Institute of General Practice and Public Health, Claudiana Bolzano, Bolzano, Italy; ^5^Department of Neurology, Medical University of Innsbruck, Innsbruck, Austria; ^6^Tyrolean Federal Institute for Integrated Care, Innsbruck, Austria; ^7^Institute of General Medicine, Medical University of Innsbruck, Innsbruck, Austria

**Keywords:** COVID-19, SARS-CoV-2, depression, anxiety, mental stress, neurocognitive, long COVID, machine learning

## Abstract

**Background:**

Coronavirus Disease-19 (COVID-19) convalescents are at risk of developing a *de novo* mental health disorder or worsening of a pre-existing one. COVID-19 outpatients have been less well characterized than their hospitalized counterparts. The objectives of our study were to identify indicators for poor mental health following COVID-19 outpatient management and to identify high-risk individuals.

**Methods:**

We conducted a binational online survey study with adult non-hospitalized COVID-19 convalescents (Austria/AT: *n* = 1,157, Italy/IT: *n* = 893). Primary endpoints were positive screening for depression and anxiety (Patient Health Questionnaire; PHQ-4) and self-perceived overall mental health (OMH) and quality of life (QoL) rated with 4 point Likert scales. Psychosocial stress was surveyed with a modified PHQ stress module. Associations of the mental health and QoL with socio-demographic, COVID-19 course, and recovery variables were assessed by multi-parameter Random Forest and Poisson modeling. Mental health risk subsets were defined by self-organizing maps (SOMs) and hierarchical clustering algorithms. The survey analyses are publicly available (https://im2-ibk.shinyapps.io/mental_health_dashboard/).

**Results:**

Depression and/or anxiety before infection was reported by 4.6% (IT)/6% (AT) of participants. At a median of 79 days (AT)/96 days (IT) post-COVID-19 onset, 12.4% (AT)/19.3% (IT) of subjects were screened positive for anxiety and 17.3% (AT)/23.2% (IT) for depression. Over one-fifth of the respondents rated their OMH (AT: 21.8%, IT: 24.1%) or QoL (AT: 20.3%, IT: 25.9%) as fair or poor. Psychosocial stress, physical performance loss, high numbers of acute and sub-acute COVID-19 complaints, and the presence of acute and sub-acute neurocognitive symptoms (impaired concentration, confusion, and forgetfulness) were the strongest correlates of deteriorating mental health and poor QoL. In clustering analysis, these variables defined subsets with a particularly high propensity of post-COVID-19 mental health impairment and decreased QoL. Pre-existing depression or anxiety (DA) was associated with an increased symptom burden during acute COVID-19 and recovery.

**Conclusion:**

Our study revealed a bidirectional relationship between COVID-19 symptoms and mental health. We put forward specific acute symptoms of the disease as “red flags” of mental health deterioration, which should prompt general practitioners to identify non-hospitalized COVID-19 patients who may benefit from early psychological and psychiatric intervention.

**Clinical Trial Registration:**

[ClinicalTrials.gov], identifier [NCT04661462].

## Background

Prevalence of mental health disorders rose during the Coronavirus Disease-19 (COVID-19) pandemic in the general population from 4% in 2006 (1) to 20% for depression and from 5% in 2008 (2) to 19% for anxiety as of March 2020 (3). The mental health deterioration following COVID-19 was described primarily for hospitalized subjects (4). The frequency of depression or anxiety (DA) following inpatient COVID-19 treatment was estimated at approximately 25% at 5–12 months post-infection (5–7). A real-world analysis of 62,354 COVID-19 in- and outpatients at 14–90 days follow-up revealed the overall incidence of psychiatric conditions of 18.1% [95% CI: 17.6–18.6], out of which 5.8% [5.2–6.4] comprised *de novo* disorders (8). In the latter study, a pre-existing mental illness was put forward as a risk factor for severe acute respiratory syndrome coronavirus 2 (SARS-CoV-2) infection suggestive of a bi-directional relationship between psychiatric conditions and COVID-19 (8). A large, medical record-based comparison of long-term sequelae in COVID-19 and non-COVID-19 healthcare system users revealed an excess of sleep/wake- (relative risk: 14.5 [11.5–17.3]), anxiety/fear- (5.4 [3.4–7.3]), and trauma/stress-related disorders (8.9 [6.6–11.1]) in COVID-19 convalescents at 6 months after the disease onset (9). Post-acute sequelae, such as mental health symptoms, occurred in decreasing frequency in intensive care unit (ICU) patients, inpatients, and outpatients (9). However, a smaller study found that individuals treated as outpatients showed worse mental health outcomes than those treated as inpatients (10). Hence, the prevalence and especially risk factors of mental health conditions and diminished quality of life (QoL) in COVID-19 outpatients, which may be missed from large-scale medical record analyses, cross-sectional, or inpatient survivor studies, are still insufficiently characterized. Since mild ambulatory cases comprise the great majority of COVID-19 patients (11), mental health sequelae may pose a significant burden to the healthcare system. For this reason, the characteristic of risk factors and subsets of patients at particular risk of post-COVID-19 is of great importance.

Machine learning (ML) and clustering algorithms gain importance at risk profiling and patient classification in high dimensional data sets in multiple conditions, i.e., COVID-19 (7, 12–16). The Random Forest procedure employs ensembles of multiple regression or classifier tree models trained in random subsets of the data set to predict the outcome (17, 18). As such, this algorithm is resistant to over-fitting (17) and was shown to reliably deal with large, multi-parameter imbalanced medical data sets (18). It also provides variable importance measures, which allow drawing conclusions on mechanistic relationships between the outcome and specific explanatory factors (15–17, 19). Self-organizing maps (SOMs) are a class of artificial neural network algorithms that enable the reduction of dimensionality of multi-parameter data sets, classification, and clustering of observations with similar properties (20, 21).

The binational “Health after COVID-19 in Tyrol” study aimed at exploring the disease course as well as physical and mental recovery in two cohorts of non-hospitalized convalescents (12). Herein, using multi-parameter Random Forest modeling, we sought to assess the impact of demographics, socioeconomics, comorbidities, COVID-19 disease symptoms and course, and psychosocial stress on anxiety, depression, self-perceived overall mental health (OMH), and QoL. By clustering analysis with SOMs, we aimed to identify individuals at risk of worsening mental health and QoL, which may particularly benefit from early psychological and psychiatric support. Finally, we made the study results publicly available as an online dashboard^1^ (22).

## Materials and Methods

### Bioethics

The study was conducted in accordance with the Declaration of Helsinki and the European data policy. Each participant gave a digitally signed informed consent to participate. The study protocol was reviewed and approved by the institutional review boards of the Medical University of Innsbruck (AT, approval number: 1257/2020) and of the Autonomous Province of Bolzano – South Tyrol (IT: 0150701).

### Study Design and Approval

**FIGURE 1:**
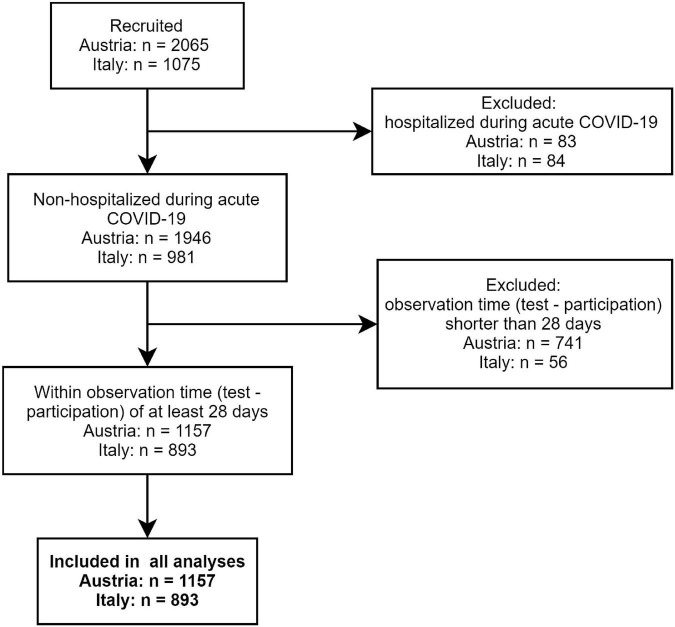
Study inclusion flow diagram.

The multi-center online survey study “Health after COVID-19 in Tyrol” (ClinicalTrials.gov: NCT04661462) was conducted between the September 30, 2020 and July 11, 2021 in two cohorts independently recruited in Tyrol/Austria (AT) and South Tyrol/Italy (IT) (12). The study inclusion criteria were residency in the study regions, age of ≥16 (AT) or ≥18 years (IT), and a laboratory-confirmed SARS-CoV-2 infection (PCR or seropositivity). Respondents with a minimum observation time of <28 days between the infection diagnosis and survey completion or hospitalized because of COVID-19 were excluded from the analysis (Figure 1). The participants were invited by a public media call (AT and IT) or by their general practitioners (IT).

### Measures, Definitions, and Data Transformation

The detailed description of the questionnaire variables is provided in Supplementary Methods, Supplementary Table 1, and by Sahanic et al. (12).

Symptoms were classified as acute complaints present during the first 2 weeks after clinical onset, sub-acute symptoms present at 2–4 weeks after clinical onset, and persistent symptoms present for ≥4 weeks (12, 15). Confusion, impaired concentration, and forgetfulness were subsumed under “neurocognitive symptoms.”

Pre-existing health conditions, such as depression/anxiety and sleep disorders, were surveyed as single items each (question: “Previous illnesses (existing or previously experienced): (1) depression/anxiety, (2) insomnia,” answers: present/absent) (12). Self-perceived OMH (question: “How do you currently estimate your mental health?”) and QoL (question: “How do you estimate your current QoL?”) were rated with a 4-point Likert scale (“excellent,” “good,” “fair,” “poor,” scored: 0, 1, 2, and 3). Anxiety and depression at the survey completion were investigated using the Patient Health Questionnaire (PHQ-4) questionnaire (anxiety: “Since the COVID 19 infection: (1) have you experienced nervousness, anxiety? (2) were you not able to stop or control worries?” depression: “Since the COVID 19 infection: (1) have you experienced little interest of satisfaction at your activities? (2) have you experienced prostration, melancholy, or hopelessness?” answers: “not at all,” “at some days,” “at more than half of the days,” “almost every day,” scored: 0, 1, 2, and 3) (12, 22, 23), with ≥3 points cutoffs for the clinical signs of depression (DPR) or anxiety (ANX). Psychosocial stress was measured with a modified 7 item PHQ stress module (questions: “How much have you felt affected since the COVID 19 infection by the following problems: (1) worries about your health? (2) Difficulties with the spouse/partner? (3) Burden of care for children parents or other relatives? (4) Stress at work or school/training? (5) Financial problems or worries? (6) Worries about your workplace? (7) Thoughts or dreams on COVID-19?”r answers: “no,” “little,” “some,” “a lot,” scored: 0, 1, 2, and 3) (22, 24), without items on weight, sexuality, and past traumatic/serious events; the item on worries/dreams was adapted to COVID-19. Substantial psychosocial stress was defined by a ≥7 points cutoff.

### Statistical Analysis

Data were analyzed with R version 4.0.5 (25, 26). Statistical significance of variable median or distribution differences between groups were determined by the Mann–Whitney (effect size: r statistic), Kruskal–Wallis (effect size: η^2^ statistic), or χ^2^ test (effect size: Cramer’s V), as appropriate. The correlation of numeric variables was investigated with Spearman ρ. Categorical variable co-occurrence was assessed by Cohen’s κ and Z test. R packages, *DescTools*, *rstatix*, *R Companion*, and *vcd*, were employed in hypothesis testing and correlation analysis. Values of *p* were corrected for multiple comparisons with the Benjamini-Hochberg method (27).

Data pre-processing prior to Random Forest modeling and clustering included minimum/maximum normalization of numeric explanatory features (Supplementary Table 1) and mental health scores. Numeric variables were not stratified prior to modeling or clustering. Random Forest models for ANX, DPR, OMH, and QoL scoring were trained, optimized, calibrated, and cross-validated (10-fold) in the AT training cohort (packages *ranger, caret*, and *qgam*) (17, 28–30) and validated in the IT collective. To account for possible effects of diagnosis – survey time, a continuous observation time variable was included in the models (Supplementary Table 1). The importance of explanatory variables in Random Forest modeling was determined by the unbiased difference in mean squared error (ΔMSE) (19, 29). The set of common influential variables from mental health and QoL scoring was determined as a common part of the top 20 most influential factors for ANX, DPR, OMH, and QoL each. Modeling of the impact of the common influential variables on mental scoring was accomplished with uni- and multivariate, age- and sex-weighted Poisson regression (12). The effects of the survey duration and observation time on mental health and QoL scoring was investigated by GAM (generalized additive modeling, package mgcv, cubic spline transformation of the independent variable). Clusters of the training AT cohort individuals were defined with the self-organized map procedure (SOM, 13 × 13 unit hexagonal grid, Manhattan distance, package *Kohonen*) and subsequent hierarchical clustering (Ward D2 algorithm, Manhattan distance) (20, 21, 31). Assignment of the test IT cohort participants to the clusters was done with the k-nearest neighbor label propagation algorithm (12, 32). Details of statistical analysis are provided in Supplementary Methods.

## Results

### Sociodemographic and Clinical Characteristics of the Study Cohorts

In total, 1,157 questionnaires in the AT and 893 in the IT cohort were analyzed (Figure 1). The observation time defined as the time period between the survey completion and the SARS-CoV-2 infection diagnosis was 79 (median, interquartile range [IQR]: 40–180) and 96 days (median, IQR: 60–140) in the AT and IT collective, respectively (Table 1). Detailed characteristics of the cohorts were reported by Sahanic et al. (12). In brief, study participants were predominantly working-age (31–65 years: AT: 71.9%, IT: 77.8%), women (AT: 65.1%, IT: 68.3%), and actively employed (>80%). Pre-existing co-morbidities were declared by 41.2% (IT) and 49.7% (AT) of participants. DA (AT: 6%, IT: 4.6%) and sleep disorders (AT: 4.6%, IT: 4%) before COVID-19 were reported by roughly 1 of 20 respondents (Table 1). Notably, the overlap between the pre-existing depression/anxiety and sleep problems was only minute (AT: Cohen’s κ = 0.21 [95% CI: 0.1–0.31], IT: κ = 0.17 [0.048–0.3]). The collectives significantly differed in language, education, employment structure, completion time, and the time interval between the diagnosis and survey completion (Table 1).

**TABLE 1:** Baseline characteristics of the study cohorts.

Variable	AT^1^	IT^1^	Test^2^	pFDR^3^	Effect size^4^
Survey completion	Fall 2020: 63% (734) winter/spring 2021: 37% (423) Complete: *n* = 1157	Fall 2020: 4.4% (39) winter/spring 2021: 96% (854) Complete: *n* = 893	χ^2^	*p* < 0.001	*V* = 0.6
Time between survey and diagnosis	Median = 79 [IQR: 40 – 180] Range: 28 – 400 Complete: *n* = 1157	Median = 96 [IQR: 60 – 140] Range: 28 – 390 Complete: *n* = 893	Mann–Whitney	*p* < 0.001	*r* = 0.12
Sex	Female: 65% (753) Male: 35% (404) Complete: *n* = 1157	Female: 68% (610) Male: 32% (283) Complete: *n* = 893	χ^2^	ns (*p* = 0.19)	*V* = 0.034
Age	Median = 43 [IQR: 31 – 53] Range: 16 – 94 Complete: *n* = 1156	Median = 45 [IQR: 35 – 55] Range: 18 – 95 Complete: *n* = 891	Mann–Whitney	*p* = 0.0041	*r* = 0.069
	Up to 30 years: 22% (259) 31 – 65 years: 72% (831) >65 years: 5.7% (66) Complete: *n* = 1156	Up to 30 years: 17% (148) 31 – 65 years: 78% (693) >65 years: 5.6% (50) Complete: *n* = 891	χ^2^	*p* = 0.0082	*V* = 0.073
Education	Secondary: 44% (505) Apprenticeship: 14% (164) Elementary: 3.6% (41) Tertiary: 38% (444) Complete: *n* = 1154	Secondary: 64% (575) Apprenticeship: 0% (0) Elementary: 0.22% (2) Tertiary: 35% (315) Complete: *n* = 892	χ^2^	*p* < 0.001	*V* = 0.31
Employment status	Employed: 81% (939) Unemployed: 9.4% (109) Leave: 1.9% (22) Retired: 7.5% (87) Complete: *n* = 1157	Employed: 82% (728) Unemployed: 8.5% (76) Leave: 1.8% (16) Retired: 8.2% (73) Complete: *n* = 893	χ^2^	ns (*p* = 0.88)	*V* = 0.02
Smoking history	Never: 60% (690) Former: 31% (361) Active: 9.2% (106) Complete: *n* = 1157	Never: 66% (588) Former: 24% (215) Active: 10% (90) Complete: *n* = 893	χ^2^	*p* = 0.004	*V* = 0.079
Number of co-morbidities	Absent: 50% (582) 1: 29% (332) 2: 12% (142) 3 and more: 8.7% (101) Complete: *n* = 1157	Absent: 59% (525) 1: 25% (219) 2: 11% (102) 3 and more: 5.3% (47) Complete: *n* = 893	χ^2^	*p* < 0.001	*V* = 0.095
Daily medication	Absent: 59% (688) 1 – 4 drugs: 38% (440) 5 drugs and more: 2.5% (29) Complete: *n* = 1157	Absent: 73% (649) 1 – 4 drugs: 26% (231) 5 drugs and more: 1.5% (13) Complete: *n* = 893	χ^2^	*p* < 0.001	*V* = 0.14
Depression/anxiety before COVID-19	DA-: 94% (1088) DA+: 6% (69) Complete: *n* = 1157	DA-: 95% (852) DA+: 4.6% (41) Complete: *n* = 893	χ^2^	ns (*p* = 0.27)	*V* = 0.03
Sleep disorders before COVID-19	4.6% (53) Complete: *n* = 1157	4% (36) Complete: *n* = 893	χ^2^	ns (*p* = 0.66)	*V* = 0.013
Bruxism	7.2% (83) Complete: *n* = 1157	5.3% (47) Complete: *n* = 893	χ^2^	ns (*p* = 0.14)	*V* = 0.039
BMI before COVID-19	Normal: 56% (648) Overweigth: 28% (327) Obesity: 15% (175) Complete: *n* = 1150	Normal: 65% (570) Overweigth: 26% (231) Obesity: 9.1% (80) Complete: *n* = 881	χ^2^	*p* < 0.001	*V* = 0.1
Hypertension	11% (130) Complete: *n* = 1157	9.4% (84) Complete: *n* = 893	χ^2^	ns (*p* = 0.27)	*V* = 0.03
Cardiovascular disease	2.9% (34) Complete: *n* = 1157	2.9% (26) Complete: *n* = 893	χ^2^	ns (*p* = 1)	*V* = 8e-04
Pulmonary disease	4.1% (48) Complete: *n* = 1157	2.6% (23) Complete: n = 893	χ^2^	ns (p = 0.12)	V = 0.043
Hay fever/allergy	18% (208) Complete: n = 1157	11% (102) Complete: *n* = 893	χ^2^	*p* < 0.001	*V* = 0.091
>2 respiratory infections per year	4.4% (51) Complete: *n* = 1157	2.9% (26) Complete: *n* = 893	χ^2^	ns (*p* = 0.14)	*V* = 0.039
>2 bacterial infections per year	3.9% (45) Complete: *n* = 1157	1.3% (12) Complete: *n* = 893	χ^2^	*p* = 0.0021	*V* = 0.077

*^1^For categorical variables: percentage of the complete answers (n individuals). AT: Austria/Tyrol cohort, IT: Italy/South Tyrol cohort.*

*^2^Statistical test used to compare differences in median values (numeric variables) or distribution (categorical variables) for the AT vs. IT comparison.*

*^3^Test values of p for the AT vs IT difference corrected for multiple comparisons with Benjamini-Hochberg (FDR) method, ns: not significant.*

*^4^Effect size of the AT vs. IT difference: Wilcoxon r for numeric variables or Cramer’s V for categorical variables.*

The percentage of asymptomatic cases ranged between 8.3% (AT) and 12.3% (IT) (Table 2). Respondents declared a median of 13 complaints (out of 44 features queried, IQR: AT: 9–18, IT: 7–18) present in the first 2 weeks after clinical onset. Persistent symptoms lasting for ≥28 days (12, 15) were discerned in 47.6% (AT) and 49.3% (IT). Roughly half of the participants suffered from acute neurocognitive symptoms (AT: 48%, IT: 50.4%), such as memory or concentration deficits or confusion, in 18.2% (AT) and 22.6% (IT) at least one persistent neurocognitive symptom was present. Self-perceived complete convalescence was reported by 54% (AT) and 63% (IT) of the respondents. The median loss of physical performance following COVID-19 was 13% in the AT (IQR: 1–26%) and 11% in the IT collective (IQR: 0–25%) (Table 2).

**TABLE 2:** Characteristics of the course of SARS-CoV2 infection and convalescence in the study cohorts.

Variable	AT^1^	IT^1^	Test^2^	pFDR^3^	Effect size^4^
SARS-CoV2 outbreak	Spring 2020: 27% (309) Summer/fall 2020: 68% (789) Winter/spring 2021: 5.1% (59) Complete: *n* = 1157	Spring 2020: 16% (144) Summer/fall 2020: 54% (484) Winter/spring 2021: 30% (265) Complete: *n* = 893	χ^2^	*p* < 0.001	*V* = 0.34
Acute COVID-19 symptoms	92% (1060) Complete: *n* = 1156	88% (782) Complete: *n* = 892	χ^2^	*p* = 0.0067	*V* = 0.066
Number of acute symptoms	Median = 13 [IQR: 9 – 18] Range: 0 – 42 Complete: *n* = 1156	Median = 13 [IQR: 7 – 18] Range: 0 – 39 Complete: *n* = 892	Mann–Whitney	ns (*p* = 0.13)	*r* = 0.038
Number of acute neurocognitive symptoms	Median = 1 [IQR: 0 – 2] Range: 0 – 3 Complete: *n* = 1157	Median = 0 [IQR: 0 – 2] Range: 0 – 3 Complete: *n* = 893	Mann–Whitney	ns (*p* = 0.66)	*r* = 0.011
	0: 50% (574) 1: 20% (236) 2: 17% (197) 3: 13% (150) Complete: *n* = 1157	0: 52% (464) 1: 14% (127) 2: 16% (146) 3: 17% (156) Complete: *n* = 893	χ^2^	*p* < 0.001	*V* = 0.095
Persistent COVID-19 symptoms	48% (550) Complete: *n* = 1156	49% (440) Complete: *n* = 892	χ^2^	ns (*p* = 0.52)	*V* = 0.017
Number of persistent symptoms	Median = 0 [IQR: 0 – 3] Range: 0 – 34 Complete: *n* = 1156	Median = 0 [IQR: 0 – 3] Range: 0 – 29 Complete: *n* = 892	Mann–Whitney	ns (*p* = 0.56)	*r* = 0.015
Number of persistent neurocognitive symptoms	Median = 0 [IQR: 0 – 0] Range: 0 – 3 Complete: *n* = 1157	Median = 0 [IQR: 0 – 0] Range: 0 – 3 Complete: *n* = 893	Mann–Whitney	*p* = 0.0067	*r* = 0.065
	0: 82% (946) 1: 7.3% (84) 2: 7.8% (90) 3: 3.2% (37) Complete: *n* = 1157	0: 77% (691) 1: 5.6% (50) 2: 9.6% (86) 3: 7.4% (66) Complete: *n* = 893	χ^2^	*p* < 0.001	*V* = 0.11
Physical performance loss	Median = 13 [IQR: 1 – 26] Range: 0 – 100 Complete: *n* = 1151	Median = 11 [IQR: 0 – 25] Range: 0 – 100 Complete: *n* = 884	Mann–Whitney	ns (*p* = 0.35)	*r* = 0.024
Complete convalescence	54% (624) Complete: *n* = 1155	63% (563) Complete: *n* = 889	χ^2^	*p* < 0.001	*V* = 0.093

*^1^Percentage of the complete answers (n individuals). AT: Austria/Tyrol cohort, IT: Italy/South Tyrol cohort.*

*^2^Statistical test used to compare differences in median values (numeric variables) or distribution (categorical variables) for the AT vs. IT comparison.*

*^3^Test value of p for the AT vs IT difference corrected for multiple comparisons with Benjamini-Hochberg (FDR) method, ns: not significant.*

*^4^Effect size of the AT vs. IT difference: Wilcoxon r for numeric variables or Cramer’s V for categorical variables.*

At the time of study completion, i.e., approximately 12 weeks post-clinical COVID-19 onset, over one-fifth of the participants rated their OMH (AT: 21.8%, IT: 24.1%) or QoL (AT: 20.3%, IT: 25.9%) as fair or poor. At this time point, anxiety (ANX) was observed in 12.4% (AT) and 19.3% (IT), DPR in 17.3% (AT) and 23.2% (IT), and substantial psychosocial stress in 21.3% (AT) and 25.6% (IT) of the respondents [Table 3 and (22)]. ANX, DPR, OMH, QoL, and stress score displayed non-normal distribution with a strong skewing toward low values (Supplementary Figure 1). Importantly, the investigated mental health and QoL rating variables were only weakly associated with the participant’s observation time (R^2^ <0.011, Supplementary Figure 2) and the total survey duration (R^2^ <0.026, Supplementary Figure 3). ANX, DRP, OMH, and QoL scores were found moderately inter-correlated, with the strongest association between anxiety and depression scoring as well as OMH and QoL rating (Supplementary Figure 4A). The highest level of co-occurrence was found for clinical DPR and ANX (AT: Cohen’s κ = 0.46, IT: κ = 0.54, Supplementary Figure 4B). Of note, a similar pattern of correlation and overlap between the investigated mental health and QoL variables was observed in the participant subsets with pre-existing DA (Supplementary Figure 5). The QoL rating as well as prevalence of ANX, DPR, and substantial stress were significantly higher in the IT than in the AT study collective, yet these differences were minor (Cramer’s *V* ≤ 0.12, *r* ≤ 0.14) [Table 3 and (22)].

**TABLE 3:** Rating of the mental health following Coronavirus Disease-19 (COVID-19) in the study cohorts.

Variable	AT^1^	IT^1^	Test^2^	pFDR^3^	Effect size^4^
Overall mental health	Poor: 3.5% (40) Fair: 18% (212) Good: 49% (562) Excellent: 30% (343) Complete: *n* = 1157	Poor: 2.9% (26) Fair: 21% (189) Good: 48% (430) Excellent: 28% (248) Complete: *n* = 893	χ^2^	ns (*p* = 0.44)	*V* = 0.039
Overall mental health score	Median = 1 [IQR: 0 – 1] Range: 0 – 3 Complete: *n* = 1157	Median = 1 [IQR: 0 – 1] Range: 0 – 3 Complete: *n* = 893	Mann–Whitney	ns (*p* = 0.29)	*r* = 0.027
Quality of life	Poor: 4.3% (50) Fair: 16% (185) Good: 51% (590) Excellent: 29% (332) Complete: *n* = 1157	Poor: 3.4% (30) Fair: 23% (201) Good: 54% (485) Excellent: 20% (177) Complete: *n* = 893	χ^2^	*p* < 0.001	*V* = 0.12
Quality of life score	Median = 1 [IQR: 0 – 1] Range: 0 – 3 Complete: *n* = 1157	Median = 1 [IQR: 1 – 2] Range: 0 – 3 Complete: *n* = 893	Mann–Whitney	*p* < 0.001	*r* = 0.1
Depression Score	Median = 1 [IQR: 0 – 2] Range: 0 – 6 Complete: *n* = 1154	Median = 1 [IQR: 0 – 2] Range: 0 – 6 Complete: *n* = 892	Mann–Whitney	*p* = 0.0082	*r* = 0.063
Depression screening-positive	17% (200) Complete: *n* = 1154	23% (207) Complete: *n* = 892	χ^2^	*p* = 0.0028	*V* = 0.073
Anxiety score	Median = 0 [IQR: 0 – 2] Range: 0 – 6 Complete: *n* = 1151	Median = 1 [IQR: 0 – 2] Range: 0 – 6 Complete: *n* = 893	Mann–Whitney	*p* < 0.001	*r* = 0.14
Anxiety screening-positive	12% (143) Complete: *n* = 1151	19% (172) Complete: *n* = 893	χ^2^	*p* < 0.001	*V* = 0.094
Psychosocial stress score	Median = 4 [IQR: 2 – 6] Range: 0 – 19 Complete: *n* = 1153	Median = 4 [IQR: 2 – 7] Range: 0 – 19 Complete: *n* = 890	Mann–Whitney	ns (*p* = 0.47)	*r* = 0.019
Substantial psychosocial stress	21% (246) Complete: *n* = 1153	26% (228) Complete: *n* = 890	χ^2^	*p* = 0.045	*V* = 0.05

*^1^Percentage of the complete answers (n individuals). AT: Austria/Tyrol cohort, IT: Italy/South Tyrol cohort.*

*^2^Statistical test used to compare differences in median values (numeric variables) or distribution (categorical variables) for the AT vs. IT comparison.*

*^3^Test value of p for the AT vs. IT difference corrected for multiple comparisons with Benjamini-Hochberg (FDR) method, ns: not significant.*

*^4^Effect size of the AT vs. IT difference: Wilcoxon r for numeric variables or Cramer’s V for categorical variables.*

### Key Factors Impacting Mental Health and Quality of Life Outcomes in Coronavirus Disease-19 Convalescents

**FIGURE 2:**
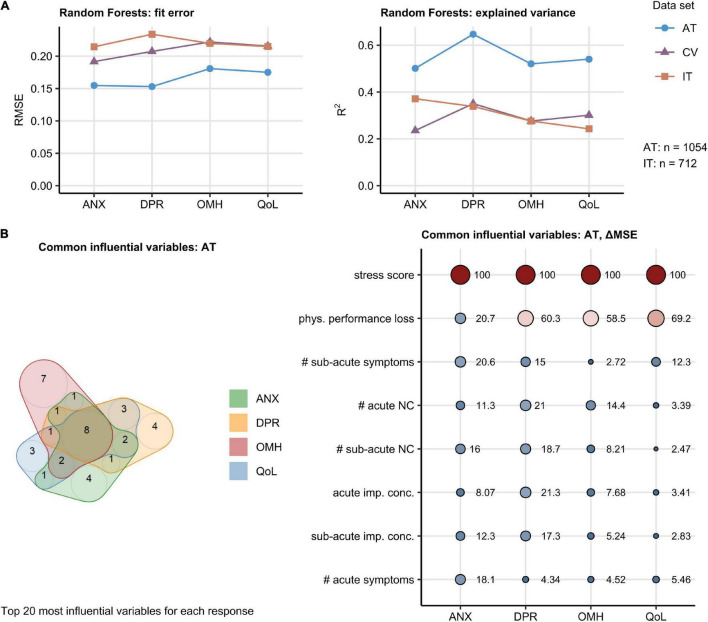
Random Forest modeling of the mental health and quality of life scoring during Coronavirus Disease-19 (COVID-19) convalescence. The effects of 201 demographic, clinical, socioeconomic, and psychosocial factors (Supplementary Table 1) on the anxiety (ANX), depression (DPR), overall mental health (OMH), and quality of life (QoL) scoring were modeled with the Random Forest technique. Numeric variables were minimum/maximum normalized prior to modeling. The models were trained and calibrated in Austria (AT) cohort, 10-fold cross-validated (CV), and their predictions validated in Italy (IT) cohort. The top 20 most influential explanatory variables were identified in the AT cohort for each mental health and life quality score by unbiased ΔMSE statistic (Supplementary Figures 6–9). The numbers of complete observations are indicated in **(A)**. **(A)** Random Forest model performance measured by root mean squared error (RMSE) and the fraction of explained variance in mental health and quality of life scoring expressed as *R*^2^. **(B)** Identification of common influential explanatory variables. Left: overlap in the top 20 most influential explanatory variables presented in a quasi-proportional Venn plot. Right: ΔMSE statistics for the most influential explanatory statistics shared by all responses, point size and color corresponds to the ΔMSE value. NC: neurocognitive symptoms, imp. conc.: impaired concentration, phys.: physical, #: number of.

We sought to investigate how the broad set of 201 surveyed demographic, socioeconomic, medical history, COVID-19 course, and recovery parameters (Supplementary Table 1) affects the minimum/maximum-normalized rating of anxiety, depression, self-perceived OMH, and QoL. To this end, Random Forest models were trained, optimized, and calibrated in the AT collective (17, 28–30). Such models demonstrated good performance with the training AT data (root mean squared error [RMSE]: 0.15–0.18) and moderate-to-good accuracy in the validation IT cohort (RMSE: 0.21–0.23). The amount of explained variance was roughly twice as large in the AT cohort (pseudo-R^2^: 0.50–0.65) as in the validation IT data set (pseudo-R^2^: 0.24–0.37) (Figure 2A and Supplementary Figures 6–9).

**FIGURE 3:**
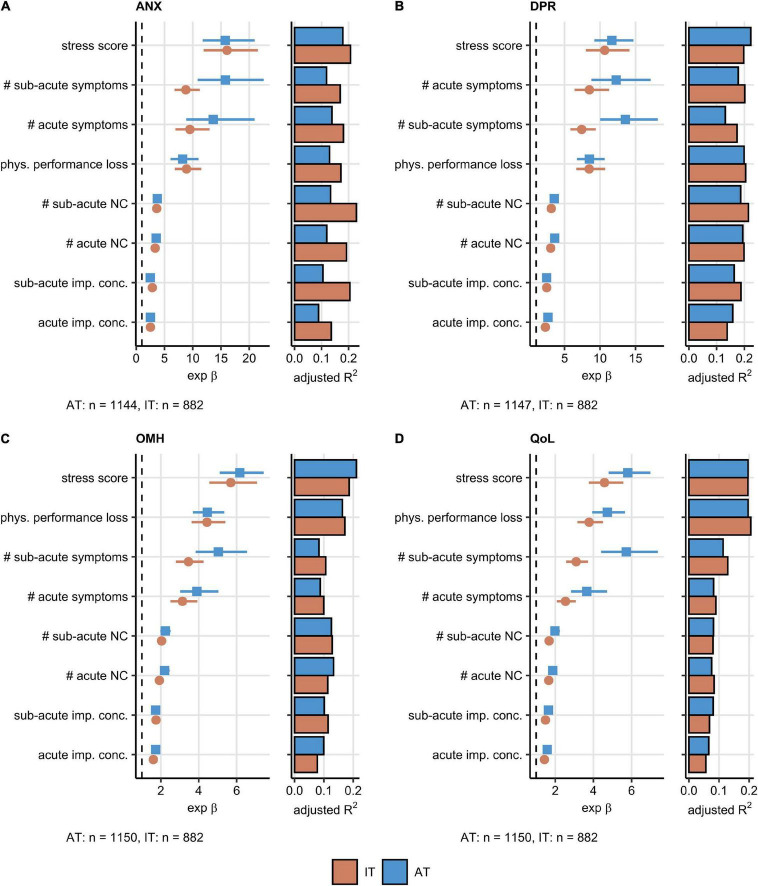
Association of the most influential factors with the mental health readouts investigated by univariable modeling. Association of the most influential factors for the mental health and quality of life scoring (Figure 2B) with the anxiety (ANX) **(A)**, depression (DPR) **(B)**, overall mental health (OMH) **(C)**, and quality of life (QoL) **(D)** rating was investigated by univariable, age- and sex-weighted Poisson regression (Supplementary Table 2). Numeric variables were minimum/maximum normalized prior to modeling. Exponent β estimate values with 95% *Cis* presented as Forest plots. Explained variance fraction estimated by adjusted R^2^ is presented in adjunct bar plots. The numbers of complete observations are shown under the plots. AT: Austria, IT: Italy. NC: neurocognitive symptoms, imp. conc.: impaired concentration, phys.: physical, #: number of.

Psychosocial stress rating and percentage of physical performance following COVID-19 were found to affect the ANX, DPR, OMH, and QoL scoring to the greatest extent in the AT cohort. The set of the 20 most important factors for each investigated response also included the total number of acute, sub-acute, and persistent COVID-19 symptoms as well as the number of specific neurocognitive symptoms. DA before COVID-19 impacted substantially the ANX, DPR, and OMH rating, pre-existing sleep disorders were found to be an influential factor for the DPR and OMH scores (Supplementary Figures 6–9). A total of eight highly influential explanatory variables were shared by the ANX, DPR, OMH, and QoL rating and included psychosocial stress, physical performance loss, acute and sub-acute symptom burden, counts of acute and sub-acute neurocognitive symptoms, as well as concentration deficits during acute and sub-acute COVID-19 (Figure 2B). In multi-variate Poisson modeling, this influential parameter set was associated with 22–37% explained variability in the mental health and QoL scoring both in the AT and IT collective (Supplementary Figure 10). Psychosocial stress, acute concentration deficits, symptom burden, and physical performance impairment are the strongest single explanatory features both in univariate and multivariate Poisson modeling of the ANX, DPR, OMH, and QoL rating following COVID-19 (Figures 3A–D, Supplementary Figure 10, and Supplementary Table 2).

Of note, the performance of the Random Forest models of ANX, DPR, OMH, and QoL modeling developed in the entire AT cohort was similar in the subsets of participants with and without pre-existing DA (Supplementary Figure 11).

### Acute and Sub-Acute Neurocognitive Symptoms and Polysymptomatic Coronavirus Disease-19 Define the Subjects at Risk of Poor Mental Health

Next, we explored whether the set of the eight most influential factors impacting the mental health and QoL in the AT or IT cohort (Figure 2) may be applied to identify convalescents at particular risk of mental health deterioration following COVID-19.

**FIGURE 4:**
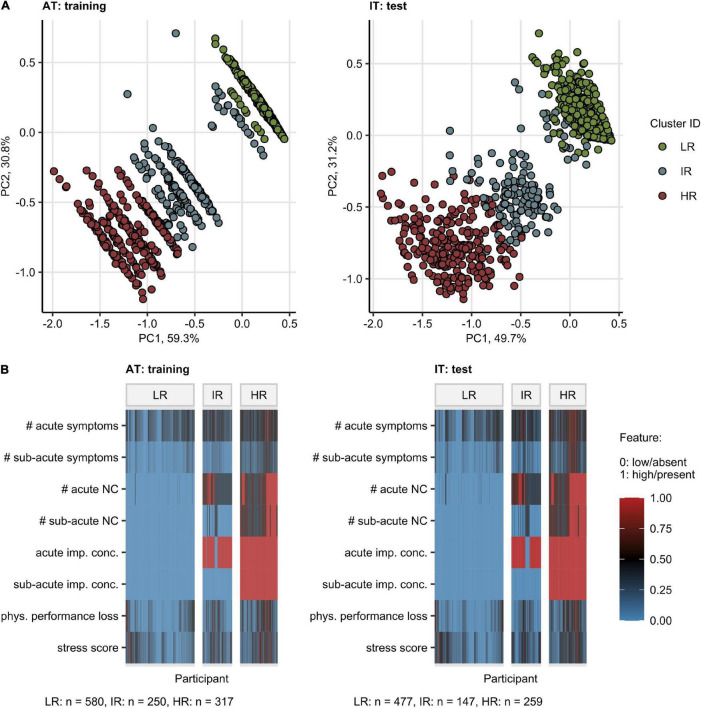
Clustering of the study participants by the most influential factors affecting the mental health and quality of life scoring. Study participants were assigned to the Low Risk (LR), Intermediate Risk (IR), and High Risk (HR) subsets by clustering in respect to the most influential factors for the mental health and quality of life scoring (Figure 2B). Numeric variables were minimum/maximum normalized prior to modeling. The procedure in the training Austria (AT) cohort involved the self-organizing map (SOM, 13 13 hexagonal grid, Manhattan distance between participants) and the hierarchical clustering (Ward D2 method, Manhattan distance between the SOM nodes) algorithms. Assignment of Italy (IT) cohort participants to the clusters was accomplished by the k-nearest neighbors classification. The numbers of participants assigned to the clusters are presented in **(B)**. **(A)** Cluster assignment of the participants in the 3-dimensional principal component (PC) analysis score plot. The first two components are shown. Percentages of the data set variance associated with the particular PC are presented in the plot axes. **(B)** Heat map of the minimum/maximum-normalized clustering features. NC: neurocognitive symptoms, imp. conc.: impaired concentration, phys.: physical, #: number of.

By an SOM and hierarchical clustering (20, 31), three participant subsets, termed “Low Risk” (LR), “Intermediate Risk” (IR), and “High Risk” (HR) Mental Health Risk Clusters, were defined in the training AT cohort and validated in the IT collective with high consistency (between-cluster to total variance ratio, AT: 0.90, IT: 0.90) (Figure 4A and Supplementary Figure 12). The primary hallmarks of the IR and HR subsets were highly frequent acute neurocognitive symptoms and, in particular, impaired concentration as well as polysymptomatic acute COVID-19. The HR subset differed from the IR cluster by the presence of sub-acute neurocognitive complaints, i.e., confusion, memory, or concentration deficits beyond the first 2 weeks of the disease (Figure 4B).

**FIGURE 5:**
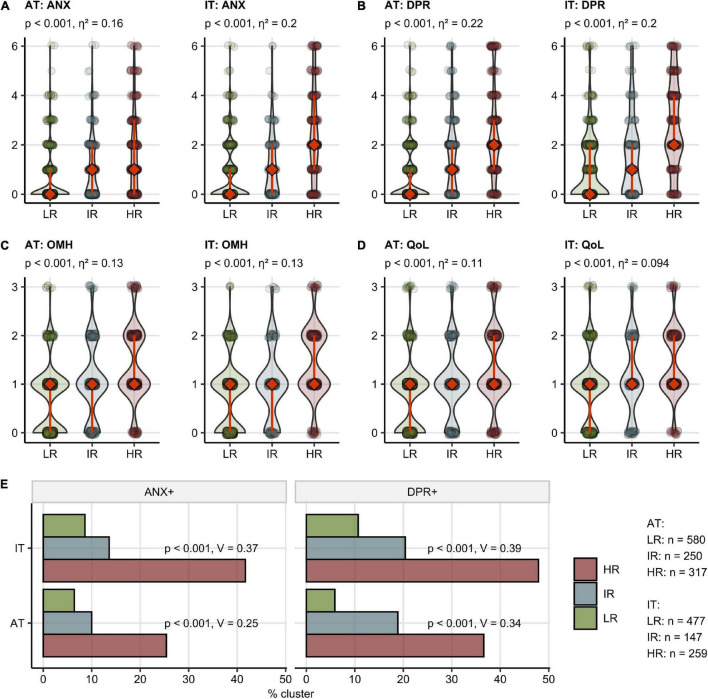
Mental health and quality of life scoring, depression and anxiety prevalence in the mental health risk clusters. Study participants were assigned to the Low Risk (LR), Intermediate Risk (IR), and High Risk (HR) subsets as presented in Figure 4. The numbers of participants assigned to the clusters are presented in **(E)**. **(A–D)** Rating of anxiety (ANX) **(A)**, depression (DPR) **(B)**, overall mental health (OMH) **(C)**, and quality of life (QoL) **(D)** in the clusters presented as violin plots, diamonds with whiskers represent medians with IQRs. Statistical significance was assessed by the Kruskal–Wallis test. *P*-values corrected for multiple testing with the Benjamini-Hochberg method and η^2^ effect size statistic values are shown in the plot captions. **(B)** Frequency of positive depression (DPR+) and anxiety (ANX+) screening in the clusters. Statistical significance was assessed by the Benjamini-Hochberg-corrected χ^2^ test, the effect size was expressed as Cramer’s V.

Notably, the HR followed by the IR group demonstrated significantly worse ANX, DPR, OMH, and QoL rating as well as higher frequencies of clinically relevant anxiety (AT: 6.4% in LR, 25.4% in HR, IT: 8.6% in LR, 41.7% in HR) and depression (AT: 5.9% in LR, 36.6% in HR, IT: 10.7% in LR, 47.9% in HR) compared with the LR cluster [Figure 5 and (22)]. In addition, the IR and HR clusters were characterized by lower frequency of self-reported complete convalescence, greater weight loss, higher levels of stress, higher symptom duration time, as well as higher frequency of acute and sub-acute fatigue, tiredness, and sleep problems as compared with the LR cluster in both AT and IT cohort (Supplementary Figures 13, 14 and Supplementary Table 3).

### Depression or Anxiety Before Coronavirus Disease-19 Is Linked to a Higher Symptom Burden and Persistence

Finally, we sought to investigate differences in the pre-COVID-19 characteristics, disease course, and recovery between the participants with and without pre-existing DA.

**FIGURE 6:**
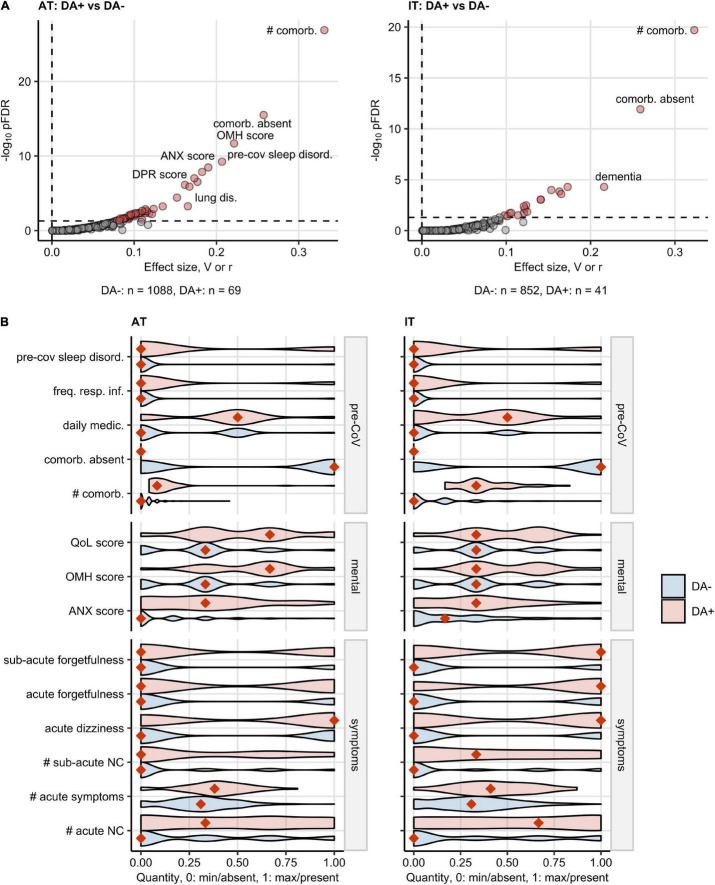
Characteristic of baseline features, COVID-19 course, and recovery in participants with pre-existing depression or anxiety. Differences in baseline characteristic, COVID-19 course, recovery, mental health, and quality of life scoring between the participants with pre-existing depression or anxiety (DA+) and the subjects without depression/anxiety history (DA–) were assessed by the χ^2^ or Mann–Whitney test in Austria (AT) and Italy (IT) cohort. The numeric variables were minimum/maximum normalized prior to modeling. The testing results were corrected from multiple testing with the Benjamini-Hochberg method (FDR: False Discovery Rate). The numbers of DA+ and DA– participants are shown in **(A)**. **(A)** Multiple testing-adjusted significance (pFDR) and effect size (categorical: Cramer’s V for categorical factors, numeric features: Wilcoxon r) for the investigated variables. Variables significantly different between DA+ and DA – are highlighted in red. **(B)** Values of the features significantly different between DA+ and DA– participants in both AT and IT collectives presented in violin plots. The numeric features were minimum/maximum normalized. Orange diamonds represent mode (categorical variables) or median values (numeric variables). pre-CoV: before COVID-19, sleep disord.: sleep disorder, freq. resp. inf.: >2 respiratory infections per yes before COVID-19, daily medic.: number of drugs taken daily, comorb.: comorbidities, #: number of, QoL: quality of life, OMH: overall mental health, ANX: anxiety, NC: neurocognitive symptoms.

In both study collectives, the DA-positive participants suffered from significantly more comorbidities, sleep disorders, and frequent respiratory infections before COVID-19 than the DA-negative respondents and, consequently, had a higher level of daily medication. Participants declaring anxiety/depression before the infection had a 20% higher median burden of overall acute COVID-19 symptoms and >30% more acute neurocognitive symptoms compared with the DA-free subset. The DA-positive participants were also more frequently affected by acute dizziness, acute, and sub-acute forgetfulness. DA before COVID-19 was also linked to a significantly worse self-perceived QoL, OMH, and a higher anxiety scoring (Figure 6 and Supplementary Table 3).

## Discussion

**FIGURE 7:**
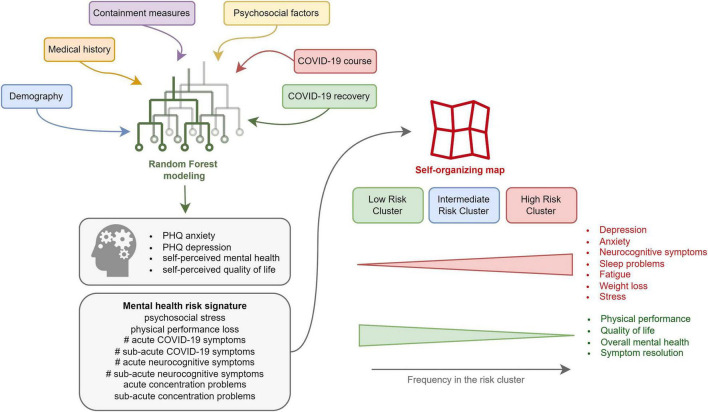
Summary of the study results.

In our binational survey, approximately 20% of non-hospitalized COVID-19 convalescents reported poor OMH, reduced QoL, or clinical DPR or ANX at about 3 months post-infection. High psychosocial stress and self-reported physical performance loss, high number of acute COVID-19 symptoms, incomplete symptom resolution within the first 2 weeks of the disease, as well as acute and sub-acute neurocognitive manifestations (impaired concentration, confusion, and forgetfulness) were identified as strong explanatory factors (Figure 7).

So far, mental health disorders following COVID-19 have been investigated primarily in hospitalized patients. Signs of at least one psychiatric sequelae (post-traumatic stress disorder [PTSD], depression, anxiety, insomnia, and obsessive-compulsive symptomatology) were discerned in 56% of inpatients at 1 month after discharge (10). Anxiety, depression, and sleep difficulties were present in approximately one-quarter of hospitalized COVID-19 individuals at the 5–12 months of follow-ups (5–7). In large-scale studies encompassing both in- and outpatients, COVID-19 was identified as an important risk factor for anxiety, stress-related, and depressive adjustment disorders (9) and mental health conditions were ascertained in nearly one-fifth of COVID-19 convalescents (8). Of note, this figure is comparable with the frequency of PHQ-4 positive anxiety (AT: 12.4%, IT: 19.3%) and depression screening (AT: 17.3, IT: 23.2%) in our study cohorts. The variability of the reported rates of depression or anxiety in COVID-19 convalescents could be explained both by the differences in assessment methods and by the differing regional containment policies reflected by the rising frequencies of mental conditions in the general population (3). This may explain the significantly higher prevalence of post-COVID-19 depression and anxiety in the IT than in the AT study cohort, despite the similar frequency of pre-existing depression or anxiety.

Our results underscore the negative impact of psychosocial stress, physical performance impairment during convalescence, acute and sub-acute neurocognitive symptoms, such as concentration and memory deficits on the mental health rating. This likely reflects a net influence of the disease itself and the pandemic management measures, such as restricted physical activity due to quarantine (33) or increased loneliness and boredom (34). Mental health status was also investigated during past outbreaks of infectious diseases, such as Ebola (35) or H1N1 influenza (36), however, it has never been evaluated as rigorously as during the SARS-CoV-2 pandemic (37). Compared to individuals who were hospitalized for seasonal influenza, COVID-19 inpatients show a higher burden of mental health problems (9), but nevertheless, it cannot unambiguously be concluded whether this is due to viral factors or associated psychosocial factors and pandemic management. Psychoneuroimmunological processes, such as low-grade inflammation and associated microglia changes, were suggested to contribute to mental health problems following COVID-19 (38, 39). Such neuropathological alterations may also provide an explanation as, why acute neurocognitive complaints posed a “red flag” of subsequent mental health deterioration in our study cohorts. Other factors, such as Vitamin D, mitochondrial dysfunction, or gut dysbiosis, might also link COVID-19 pathobiology and mental health (40). In Random Forest modeling of mental health scoring in our study collectives, impaired physical performance following COVID-19 was found to impact particularly depression, OMH, and QoL rating. A similar phenomenon was described by Evans et al. (7) in hospitalized COVID-19 patients, who linked physical impairment with poor mental health status, respiratory symptoms, fatigue, and protracted systemic inflammation. Of note, a reciprocal axis between physical performance and mental health, and especially depression, may exist since physical impairment is one of the diagnostic criteria of major depressive disorder (41).

The neurocognitive complaints during acute COVID-19 were found frequently accompanied by lower respiratory, cardiological, neurological symptoms, and sleep disorders (7, 9, 12, 15, 42–45). Such “multi-organ phenotype” of COVID-19 was found by us to be a correlate of protracted clinical recovery (12). Herein, the neurocognitive features together with the high symptom burden of acute COVID-19, fatigue, tiredness, and sleep problems hallmarked the IR and HR Mental Health Risk Clusters of the participants likely to develop a mental health condition in course of the recovery. Such “red flags” of deteriorating mental health present in the first 2 weeks of COVID-19 may be exploited for early diagnosis and psychological or psychiatric intervention.

Pre-existing depression or anxiety was reported by roughly 5% of the respondents and was linked to mental health deficits during recovery – a phenomenon known from non-COVID-19 medical conditions (46). The Random Forest models demonstrated a comparable performance in the DA-positive and -negative study participants. This suggests that the major factors determining the mental health rating following COVID-19 were likely common for individuals with and without pre-existing DA. Concomitantly, the subset with pre-existing DA was found to experience a significantly higher burden of acute symptoms as well as acute and sub-acute neurocognitive complaints. However, it needs to be clarified whether this is attributed to the observed higher level of additional co-morbidity, increased susceptibility to respiratory infections, or different perception for symptoms in the DA subsets in our study. Conspicuously, psychiatric disorders before COVID-19 were described as age- and other comorbidity-independent risk factors for SARS-CoV-2 infection (8). This may indicate that alike chronic somatic diseases, pre-existing mental health conditions may predispose the patient to more severe and polysymptomatic COVID-19.

Several mechanisms might mediate the bidirectional associations of COVID-19, depression, anxiety, and psychosocial stress (47). Protracted systemic inflammation is an important pathogenetic factor in depressive-anxious disorders during COVID-19 convalescence (7, 10, 12, 48–50). Stress being the key co-variate of poor mental health in the study collectives was proposed to modulate anti-SARS-CoV-2 immunity culminating in more severe COVID-19 (51) and to perpetuate the systemic low-grade inflammation (46, 51). Other possible mechanisms include direct viral infection of the central nervous system, neuroinflammation, microvascular thrombosis, and neurodegeneration (52). The strong association of acute neurocognitive manifestations with poor mental health scoring in our study suggests that pathobiological processes triggered likely by the pathogen and anti-SARS-CoV-2 immunity early in the disease course may contribute to the mental health deterioration. Targeted investigations of COVID-19 recovery with mental health, biochemical, and immunological readouts are missing in our current survey study, and a case-control design is needed to shed more light on this phenomenon and to entangle the mechanistic interplay between acute COVID-19 pathobiology, recovery, and mental health status (53). The prime strength of our study is the inclusion of two independently recruited cohorts differing in socioeconomic structure and national containment measures which allowed for identification and validation of common influencing factors. Furthermore, the study cohorts encompassed outpatients only insufficiently characterized so far. The most important study limitation is a possible participants’ selection bias. The majority of respondents showed good mental health before COVID-19, and it is likely that predominantly individuals with severe or persistent COVID-19 symptoms and high health-awareness completed the survey (12). Modeling of the impact of the individual observation time and the total survey duration indicated that those two potential sources of bias had only a minor effect on the rating of mental health and QoL. Notably, the observation time variable was included in the multi-parameter models (22). Despite a broad set of independent study variables, the fraction of unexplained variability of the mental health and QoL scoring was substantial, especially in the validation Italian cohort. We could speculate that a higher explanatory power may be reached by the inclusion of additional explanatory variables concerning stringency of national containment measures, socioeconomic background (family status, income, and care duties), personal attitude to the pandemic management, or impact of the outbreak on one’s lifestyle, which may drive the raising frequency of mental disorders in the non-infected population as well (3, 34).

This study underlines the importance of mental health in the follow-up care of COVID-19 individuals. Psychosocial stress, polysymptomatic disease, and neurocognitive complaints during acute COVID-19 are proposed as a risk signature of a subsequent mental disorder (Figure 7). They may prompt clinicians, i.e., general practitioners, to monitor outpatients with COVID-19 more closely for mental health deterioration and identify those who could benefit from early psychological and psychiatric intervention. Additionally, a pre-existing mental health condition may pose a risk factor of more severe COVID-19.

## Data Availability Statement

The raw data supporting the conclusions of this article will be made available by the authors, without undue reservation. Analysis of the psychosocial features is available as an online dashboard (https://im2-ibk.shinyapps.io/mental_health_dashboard). The complete R analysis pipeline is available at https://github.com/PiotrTymoszuk/mental-health-after-COVID-19.

## Ethics Statement

The studies involving human participants were reviewed and approved by the Institutional Review Board of the Medical University of Innsbruck (approval number: 1257/2020) and the Institutional Review Board of the Autonomous Province of Bolzano – South Tyrol (approval number: 0150701). The patients/participants provided their digital written informed consent to participate in this study.

## Author Contributions

AH, AP, BS-U, CW, GP, GW, HB, JL-R, KH, MG, RB-W, RH, SK, SS, and VR designed the study. KH, DA, SS, AP, VR, MG, AB, KK, TS, IT, BP, CW, HB, and GP collected the data. KH, PT, and DA performed data analysis. PT, DA, KH, RH, BS-U, and JL-R interpreted the data. PT, DA, KH, BS-U, and JL-R wrote the manuscript. All authors critically reviewed the final version of the manuscript.

## Conflict of Interest

PT owns Data Analytics as a Service Tirol and has received an honorarium from the ‘Health after COVID-19 in Tyrol’ study team from the Medical University of Innsbruck and Claudiana Bolzano for the study data management, curation and analysis, and minor manuscript work. The remaining authors declare that the research was conducted in the absence of any commercial or financial relationships that could be construed as a potential conflict of interest.

## Publisher’s Note

All claims expressed in this article are solely those of the authors and do not necessarily represent those of their affiliated organizations, or those of the publisher, the editors and the reviewers. Any product that may be evaluated in this article, or claim that may be made by its manufacturer, is not guaranteed or endorsed by the publisher.

## Footnotes

1
https://im2-ibk.shinyapps.io/mental_health_dashboard/
