# Supplementary material for: Who Is at Risk of Poor Mental Health Following Coronavirus Disease-19 Outpatient Management?
Source: Front Med (Lausanne). 2022 Mar 14;9:792881. doi: 10.3389/fmed.2022.792881 (PMC8964263; doi:10.3389/fmed.2022.792881)
Supplement: Supplementary file 1 [file Data_Sheet_1.PDF]

Who is at risk of poor mental health following COVID-19 outpatient management?

Supplementary Material

Supplementary Methods

Study design and participants

The multi-center bi-national online survey study ‘Health after COVID-19 in Tyrol’ (ClinicalTrials.gov: NCT04661462) was conducted between the 30th September 2020 and 11th July 20221 in two independently recruited cohorts in Tyrol/Austria (AT) and South Tyrol/Italy (IT) (1). The study cohorts encompassed residents of the study regions aged ≥ 16 (AT) or ≥ 18 years (IT) who experienced a laboratory-confirmed SARS-CoV-2 infection (PCR or seropositivity). The respondents with a minimum observation time of <28 days between the infection diagnosis and survey completion or hospitalized because of COVID-19 were excluded from the analysis. The scheme of study and analysis enrollment is depicted in **Figure 1**. The participants were invited by a public media call (both cohorts) or by their general practitioners (IT).

The study was conducted in accordance with the Declaration of Helsinki as well as the national and European data policies. Each participant gave a digitally signed informed consent to participate. The study protocol was approved by the institutional review boards of the Medical University of Innsbruck (AT, approval number: 1257/2020) and of the Autonomous Province of Bolzano - South Tyrol province (IT, 0150701).

Measures, definitions and data transformation

The detailed description of the questionnaire is provided by Sahanic et al (1). In brief, dates of the study completion and SARS-CoV2 infection diagnosis, observation time (a time interval between the positive SARS-CoV-2 test and the survey completion), data on biometry (weight, height), demographics (age, sex, body mass index/BMI), pre-existing co-morbidities, socioeconomic status (residence region, mother tongue, employment status and profession), smoking history and COVID-19 relevant medication, symptom duration (44 symptoms), symptomatic therapy and course of SARS-CoV-2 infection, recovery duration and status (self-perceived complete recovery, percent physical performance loss, new medication or conditions following COVID-19) as well as mental health and psychosocial stress following the disease were queried. The complete list of features

analyzed in the current report is presented in **Supplementary Table S1** and the baseline characteristic of the study collectives is shown in **Table 1**.

Self-reported COVID-19 symptoms were retrospectively assigned to the following duration classes: absent, present for 1 – 3 days, ≤ 1 week, ≤ 2 weeks, ≤ 4 weeks, ≤ 3 months, ≤ 6 months and > 6 months. Symptoms were classified as acute complaints present during the first 2 weeks after clinical onset, as sub-acute complaints when present at 2 - 4 weeks after clinical onset and persistent symptoms present for ≥ 4 weeks (1). Confusion, impaired concentration and forgetfulness were classified as 'neurocognitive symptoms'.

Depression/anxiety before SARS-CoV2 infection, pre-existing sleep disorders, acute COVID-19 perception (common cold-, influenza-, gastroenteritis-like or unique/not experienced before), symptom relapse, complete convalescence, rehabilitation need and percent physical performance loss following COVID-19 were surveyed as single question items each.

Self-perceived overall mental health (OMH) and quality of life (QoL) were assessed as single questions ('excellent', 'good', 'fair', 'poor', scored: 0, 1, 2, 3). Anxiety/depression following COVID-19 at time of study completion were investigated using PHQ-4 module (two questions each, possible answers: 'never', 'some days', 'over 50% of days', 'almost every day', scoring: 0, 1, 2, 3 points). Clinical signs of depression (DPR) or anxiety (ANX) were defined with the cutoffs of ≥ 3 point sum (2). Psychosocial stress was measured with a modified 7 item (answers: 'no', 'little', 'some', 'a lot', scored: 0, 1, 2, 3) PHQ stress module (3–6), without items on weight, sexuality and past traumatic/serious events; the item on worries/dreams was adapted to COVID-19. Substantial psychosocial stress was defined by a ≥ 7 point cutoff.

Statistical analysis

Data transformation, visualization, descriptive statistic and hypothesis testing

The study variables were transformed, analyzed and visualized with R version 4.0.5 with *tidyverse* (7,8) and *cowplot* (9).

For categorical variables, numbers and percents of complete answers are presented. As most of the analyzed numeric features had a discrete or non-normal distribution as checked by Shapiro-Wilk test (**Supplementary Figure S1**), medians, interquartile ranges (IQR) and feature ranges are presented. To compare differences in distribution of categorical features, χ^2 test was applied. To assess significance of differences in numeric variables between groups, U or Kruskal-Wallis test was used, as appropriate. Effect size for categorical variable differences was expressed as Cramer's V (function *CramerV()*, package *DescTools*). Effect size for numeric variables was expressed as Wilcoxon e (two-factor comparisons, function *wilcox_effsize()*, package *rstatix*) or η^2 (function *kruskal_effsize()*, package *rstatix*). Correlation of numeric variables was assessed by Spearman test (function *cor.test()*, package *stats*). Co-occurrence of two categorical variables was expressed as

Cohen's kappa statistics (function *Kappa()*, package *vcd*) (10), whose significance was assessed by Z test. P values were corrected for multiple comparisons with Benjamini-Hochberg method (11). The descriptive statistic and testing was accomplished with an in-house developed R package (ExDA, https://github.com/PiotrTymoszuk/ExDA).

Data pre-processing prior to Random Forest, Poisson modeling and clustering included minimum/maximum normalization of numeric explanatory features (**Supplementary Table 1**) and mental health scores. Numeric variables were not stratified prior to modeling or clustering.

Random Forest modeling of mental health and quality of life scoring

Multi-parameter Random Forest regression models (12) for the scoring of ANX, DPR, OMH and QoL following COVID were trained in the Austria cohort with a set of 201 demographic, clinical, socioeconomic and psychosocial factors (**Supplementary Table 1**). Of note, to account for possible recall bias of acute COVID-19 symptoms and effects of convalescence time on the scoring and frequency of clinical signs of mental health disorders, the numeric observation time variable was included in the Random Forest modeling procedure. The models were trained and cross-validated using *caret* package (function *train()*, method: 'ranger') (13,14). The data pre-processing included minimum/maximum normalization of numeric explanatory variables. Optimization of the entry model parameters ('tuning') was accomplished by 10-fold cross-validation with root mean squared error (RMSE) as the summary statistic (the tuned paramater set: splitrule = 'variance', mtry = 200, min.node.size = 40). The Random Forest models were calibrated in the post-hoc manner by the quantile GAM procedure (function *qgam()*, package *qgam*, quantiles: 0.6 for ANX, 0.55 for DPR, 0.5 for OMH and 0.5 for QoL) (15). Finally, the model predictions were verified in cross-validation and the Italy test collective. The model prediction fit to the actual scoring was assessed by RMSE and pseudo-R^2 statistics (**Supplementary Figures S6 - S9**). The model prediction, validation and visualization tasks were accomplished with an in-house developed R package (caretExtra, https://github.com/PiotrTymoszuk/caretExtra).

To discern the features with the largest effect on the ANX, DPR, OMH and QoL scoring each, differences in the unbiased mean squared error (*Δ*MSE, *ranger()* parameter importance = 'impurity_corrected') (16) associated with the model terms were extracted with *varImp()* function from *caret* package. With this procedure, top 20 most influential explanatory variables for each response were identified (**Supplementary Figures S6 - S9**). The overlap between such highly influential factors was presented as quasi-proportional Venn plots in **Figure 2B** (function *plotVenn()*, package *nVennR*) (17). The set of 8 highly influential parameters shared by the ANX, DPR, OMH and QoL models was utilized in further univariate and clustering analyses.

Univariable and multi-paramater GAM and Poisson modeling

Association of the observation time (survey - SARS-CoV-2 infection diagnosis, **Supplementary Figure S2**) and of the survey time (**Supplementary Figure S3**) with the ANX, DPR, OMH and QoL scoring was investigated by univariate Poisson generalized additive modeling (GAM, base: cubic splines, k = 20 base dimension) (18).

Association of the common influential factors identified by Random Forest modeling (**Figure 2B**) with the ANX, DPR, OMH and QoL scoring was assessed by age- and sex-weighted Poisson regression (generalized linear modeling, log link function). The frequency weights for the Austria/Tyrol and Italy/South Tyrol cohort were based on the age and sex distribution of COVID cases in Tyrol (19) and Italy (20), respectively (1). Significance of model estimates and their 95% confidence intervals were determined with Wald Z test. P values were corrected for multiple comparisons by Benjamini-Hochberg method (11). For complete univariable modeling results, see: **Figure 3** and **Supplementary Table 2**. To estimate the amount of deviance of ANX, DPR, OMH and QoL scoring explained by the common influential factors (**Figure 2B**), multi-parameter Poisson models were constructed. The fraction of explained deviance associated with specific model terms was investigated by sequential addition (function *anova.glm()*, package *stats*, **Supplementary 10**). The model Poisson model construction, quality control and fit assessment was accomplished with an in-house developed R package (lmqc, https://github.com/PiotrTymoszuk/lmqc).

Definition of the mental disorder risk clusters

The Austria cohort individuals were clustered in respect to the common influential factors identified by Random Forest modeling (**Figure 2B**) using a two-step combined self-organizing map (SOM) and hierarchical clustering algorithm (21,22). In the first step, the subjects were assigned to the nodes of 13 × 13 unit hexagonal grid with the Manhattan distance between the participants. The grid size was estimated with the $5 \times \sqrt{N}$ formula, where N is the number of observations (23). SOM assignment was accomplished with the tools provided by *kohonen* package and home-developed wrappers (https://github.com/PiotrTymoszuk/clustering-tools-2). The SOM training process is visualized in **Supplementary Figure S12A**. In the second step, SOM nodes were subjected to hierarchical clustering with Ward D2 method and Manhattan distance measure. The optimal cluster number (k = 3) was determined by the bend of the within sum-of-squares and visual analysis of the dendrograms (**Supplementary Figure S12BC**). The hierarchical clustering was done with the base *hclust()* function and home-developed wrappers for clustering quality control and visualization (https://github.com/PiotrTymoszuk/clustering-tools-2). To assign the Italy cohort individuals to the cluster developed in the Austria collective ('semi-supervided clustering'), a k-nearest neighbors label propagation classifier was employed (k = 5) (1,24). The robustness of clustering in the Austria and Italy cohorts was assessed by the between-cluster to total sum-of-squares ratio ('clustering variance', **Supplementary Figure S12**).

Data availability

As this study is still ongoing, the complete data will be made available on a serious request to the corresponding author and made publicly available after the completion. Analysis of the psychosocial features is available as an online *R shiny* dashboard at Mental Health after COVID-19 in Tyrol (https://im2-ibk.shinyapps.io/mental_health_dashboard/) (6). The R analysis pipeline is available at https://github.com/PiotrTymoszuk/mental-health-after-COVID-19.

Supplementary Tables

Table S1: Survey variables used for construction of random forest models. The table is available as a supplementary Excel file.

Table S2: Results of univariate Poisson modeling for the most influential mental health scoring factors.

Cohort[1]	Response[2]	Indep. variable[3]	N level[4]	N complete[5]	exp ß[6]	R^2	pFDR[7]
AT	OMH	acute imp. conc.	545	1,156	1.7 [1.6 - 1.9]	0.099	p = 1.5e-29
		sub-acute imp. conc.	319	1,156	1.7 [1.6 - 1.9]	0.100	p = 1.8e-29
		# acute symptoms		1,155	3.9 [3 - 5]	0.088	p = 2e-24
		# sub-acute symptoms		1,155	5 [3.8 - 6.5]	0.083	p = 1.1e-30
		stress score		1,153	6.2 [5.1 - 7.4]	0.210	p = 1.3e-70
		phys. performance loss		1,150	4.5 [3.7 - 5.3]	0.160	p = 7.4e-51
		# acute NC		1,156	2.2 [2 - 2.5]	0.130	p = 1.2e-38
		# sub-acute NC		1,156	2.2 [2 - 2.5]	0.130	p = 9.3e-36
	QoL	acute imp. conc.	545	1,156	1.6 [1.4 - 1.7]	0.066	p = 6.2e-21
		sub-acute imp. conc.	319	1,156	1.6 [1.5 - 1.8]	0.081	p = 1.4e-24
		# acute symptoms		1,155	3.7 [2.8 - 4.7]	0.082	p = 3.1e-22
		# sub-acute symptoms		1,155	5.7 [4.4 - 7.4]	0.110	p = 2.4e-37
		stress score		1,153	5.8 [4.8 - 7]	0.200	p = 4.6e-66
		phys. performance loss		1,150	4.7 [3.9 - 5.7]	0.200	p = 1e-56
		# acute NC		1,156	1.9 [1.7 - 2.1]	0.076	p = 2.8e-24
		# sub-acute NC		1,156	2 [1.7 - 2.2]	0.082	p = 1.8e-25
	DPR	acute imp. conc.	544	1,153	2.7 [2.3 - 3.1]	0.160	p = 2.4e-43
		sub-acute imp. conc.	319	1,153	2.5 [2.2 - 2.8]	0.160	p = 1.5e-43
		# acute symptoms		1,152	12 [8.8 - 17]	0.180	p = 1.1e-44
		# sub-acute symptoms		1,152	14 [10 - 18]	0.130	p = 3.5e-59
		stress score		1,150	12 [9.2 - 15]	0.220	p = 3.2e-79
		phys. performance loss		1,147	8.5 [6.8 - 11]	0.200	p = 3.9e-66
		# acute NC		1,153	3.6 [3.1 - 4.2]	0.190	p = 3e-55
		# sub-acute NC		1,153	3.5 [3 - 4.1]	0.190	p = 7.2e-52

Cohort[1]	Response[2]	Indep. variable[3]	N level[4]	N complete[5]	exp ß[6]	R^2	pFDR[7]
	ANX	acute imp. conc.	542	1,150	2.5 [2.1 - 3]	0.089	p = 2.6e-25
		sub-acute imp. conc.	317	1,150	2.5 [2.1 - 2.9]	0.110	p = 2.7e-29
		# acute symptoms		1,149	14 [8.8 - 21]	0.140	p = 1.3e-30
		# sub-acute symptoms		1,149	16 [11 - 23]	0.120	p = 2.2e-45
		stress score		1,148	16 [12 - 21]	0.180	p = 2e-68
		phys. performance loss		1,144	8.2 [6.1 - 11]	0.130	p = 1.3e-40
		# acute NC		1,150	3.5 [2.9 - 4.3]	0.120	p = 5.6e-34
		# sub-acute NC		1,150	3.7 [3.1 - 4.5]	0.130	p = 5.9e-37
IT	OMH	acute imp. conc.	390	891	1.6 [1.5 - 1.8]	0.078	p = 1.5e-19
		sub-acute imp. conc.	262	891	1.7 [1.6 - 1.9]	0.110	p = 3.9e-26
		# acute symptoms		890	3.1 [2.5 - 3.9]	0.100	p = 1.8e-22
		# sub-acute symptoms		890	3.5 [2.8 - 4.3]	0.110	p = 9e-29
		stress score		888	5.7 [4.6 - 7.1]	0.190	p = 8.2e-48
		phys. performance loss		882	4.4 [3.6 - 5.4]	0.170	p = 2.1e-43
		# acute NC		891	1.9 [1.7 - 2.2]	0.110	p = 8.4e-26
		# sub-acute NC		891	2 [1.8 - 2.3]	0.130	p = 1.9e-29
	QoL	acute imp. conc.	390	891	1.4 [1.3 - 1.6]	0.056	p = 1.4e-15
		sub-acute imp. conc.	262	891	1.5 [1.4 - 1.6]	0.068	p = 1.3e-17
		# acute symptoms		890	2.5 [2.1 - 3.1]	0.090	p = 9.7e-20
		# sub-acute symptoms		890	3.1 [2.6 - 3.7]	0.130	p = 1.7e-30
		stress score		888	4.6 [3.8 - 5.6]	0.200	p = 9.2e-47
		phys. performance loss		882	3.8 [3.2 - 4.5]	0.210	p = 3.7e-44
		# acute NC		891	1.7 [1.5 - 1.8]	0.084	p = 1.4e-20
		# sub-acute NC		891	1.7 [1.5 - 1.9]	0.080	p = 4.7e-20
	DPR	acute imp. conc.	389	890	2.3 [2 - 2.6]	0.140	p = 7e-31

Cohort[1]	Response[2]	Indep. variable[3]	N level[4]	N complete[5]	exp ß[6]	R^2	pFDR[7]
		sub-acute imp. conc.	261	890	2.5 [2.2 - 2.8]	0.190	p = 4.8e-39
		# acute symptoms		889	8.5 [6.4 - 11]	0.200	p = 3.2e-44
		# sub-acute symptoms		889	7.4 [5.8 - 9.4]	0.170	p = 3.9e-53
		stress score		888	11 [8 - 14]	0.200	p = 5e-52
		phys. performance loss		882	8.5 [6.7 - 11]	0.200	p = 2.5e-59
		# acute NC		890	3 [2.6 - 3.5]	0.200	p = 4e-41
		# sub-acute NC		890	3.1 [2.7 - 3.6]	0.210	p = 7.8e-45
	ANX	acute imp. conc.	390	891	2.5 [2.2 - 2.9]	0.140	p = 1.1e-30
		sub-acute imp. conc.	262	891	2.8 [2.5 - 3.3]	0.210	p = 2.8e-43
		# acute symptoms		890	9.5 [6.9 - 13]	0.180	p = 6e-41
		# sub-acute symptoms		890	8.8 [6.8 - 11]	0.170	p = 2.1e-55
		stress score		888	16 [12 - 22]	0.210	p = 2.6e-64
		phys. performance loss		882	8.9 [6.8 - 12]	0.170	p = 3.7e-53
		# acute NC		891	3.4 [2.8 - 4]	0.190	p = 1.5e-40
		# sub-acute NC		891	3.6 [3.1 - 4.2]	0.230	p = 1.4e-49

[1]AT: Austria, IT: Italy.

[2]OMH: overall mental health, QoL: quality of life, ANX: anxiety, DPR: depression.

[3]NC: neurocognitive symptoms, imp. conc.: impaired concentration, phys.: physical, #: number of.

[4]Numbers of participants positive for the factor, relavent only for categorical variables.

[5]Complete observations.

[6]Exponent model coefficient estimate with 95% confidence intervals.

[7]Significance of the model coefficient corrected for multiple testing with Benjamini-Hochberg (FDR) method.

Table S3: Variables significantly different between the participants with and without depression/anxiety history before COVID-19.

Cohort[1]	Variable	DA-negative[2]	DA-positive[2]	Test[3]	pFDR[4]	Effect size[5]
AT	Overall Mental Health Score	Median = 1 [IQR: 0 - 1]<br>Range: 0 - 3<br>Complete: n = 1088	Median = 2 [IQR: 1 - 2]<br>Range: 0 - 3<br>Complete: n = 69	Mann-Whitney	p < 0.001	r = 0.22
	Quality of Life Score	Median = 1 [IQR: 0 - 1]<br>Range: 0 - 3<br>Complete: n = 1088	Median = 2 [IQR: 1 - 2]<br>Range: 0 - 3<br>Complete: n = 69	Mann-Whitney	p < 0.001	r = 0.18
	Anxiety score	Median = 0 [IQR: 0 - 1]<br>Range: 0 - 6<br>Complete: n = 1083	Median = 2 [IQR: 1 - 3]<br>Range: 0 - 6<br>Complete: n = 68	Mann-Whitney	p < 0.001	r = 0.19
	Co-morbidity absent	54% (591)<br>Complete: n = 1088	0% (0)<br>Complete: n = 69	χ^2	p < 0.001	V = 0.26
	> 2 respiratory infections per year	3.8% (41)<br>Complete: n = 1088	14% (10)<br>Complete: n = 69	χ^2	p = 0.0012	V = 0.12
	Sleep disorders before COVID-19	3.5% (38)<br>Complete: n = 1088	22% (15)<br>Complete: n = 69	χ^2	p < 0.001	V = 0.21
	Number of co-morbidities	Median = 0 [IQR: 0 - 1]<br>Range: 0 - 11<br>Complete: n = 1088	Median = 2 [IQR: 1 - 3]<br>Range: 1 - 24<br>Complete: n = 69	Mann-Whitney	p < 0.001	r = 0.33
	Daily medication	absent: 62% (670)<br>1 - 4 drugs: 36% (394)<br>5 drugs and more: 2.2% (24)<br>Complete: n = 1088	absent: 26% (18)<br>1 - 4 drugs: 67% (46)<br>5 drugs and more: 7.2% (5)<br>Complete: n = 69	χ^2	p < 0.001	V = 0.18
	Dizziness acute COVID-19	42% (452)<br>Complete: n = 1088	58% (40)<br>Complete: n = 69	χ^2	p = 0.044	V = 0.079
	Forgetfulness acute COVID-19	29% (318)<br>Complete: n = 1088	46% (32)<br>Complete: n = 69	χ^2	p = 0.021	V = 0.088
	Forgetfulness sub-acute COVID-19	21% (230)<br>Complete: n = 1088	36% (25)<br>Complete: n = 69	χ^2	p = 0.025	V = 0.086

Cohort[1]	Variable	DA-negative[2]	DA-positive[2]	Test[3]	pFDR[4]	Effect size[5]
	Number of acute symptoms	Median = 13 [IQR: 8 - 18]<br>Range: 0 - 42<br>Complete: n = 1087	Median = 16 [IQR: 13 - 21]<br>Range: 0 - 34<br>Complete: n = 69	Mann-Whitney	p = 0.0013	r = 0.11
	Number of acute neurocognitive symptoms	Median = 0 [IQR: 0 - 2]<br>Range: 0 - 3<br>Complete: n = 1088	Median = 1 [IQR: 0 - 2]<br>Range: 0 - 3<br>Complete: n = 69	Mann-Whitney	p = 0.0045	r = 0.1
	Number of sub-acute neurocognitive symptoms	Median = 0 [IQR: 0 - 1]<br>Range: 0 - 3<br>Complete: n = 1088	Median = 0 [IQR: 0 - 2]<br>Range: 0 - 3<br>Complete: n = 69	Mann-Whitney	p = 0.0089	r = 0.094
IT	Overall Mental Health Score	Median = 1 [IQR: 0 - 1]<br>Range: 0 - 3<br>Complete: n = 852	Median = 1 [IQR: 1 - 2]<br>Range: 0 - 3<br>Complete: n = 41	Mann-Whitney	p < 0.001	r = 0.15
	Quality of Life Score	Median = 1 [IQR: 1 - 1]<br>Range: 0 - 3<br>Complete: n = 852	Median = 1 [IQR: 1 - 2]<br>Range: 0 - 3<br>Complete: n = 41	Mann-Whitney	p = 0.019	r = 0.1
	Anxiety score	Median = 1 [IQR: 0 - 2]<br>Range: 0 - 6<br>Complete: n = 852	Median = 2 [IQR: 1 - 3]<br>Range: 0 - 6<br>Complete: n = 41	Mann-Whitney	p = 0.032	r = 0.098
	Co-morbidity absent	61% (520)<br>Complete: n = 852	0% (0)<br>Complete: n = 41	χ^2	p < 0.001	V = 0.26
	> 2 respiratory infections per year	2.5% (21)<br>Complete: n = 852	12% (5)<br>Complete: n = 41	χ^2	p = 0.018	V = 0.12
	Sleep disorders before COVID-19	3.3% (28)<br>Complete: n = 852	20% (8)<br>Complete: n = 41	χ^2	p < 0.001	V = 0.17
	Number of co-morbidities	Median = 0 [IQR: 0 - 1]<br>Range: 0 - 6<br>Complete: n = 852	Median = 2 [IQR: 2 - 3]<br>Range: 1 - 5<br>Complete: n = 41	Mann-Whitney	p < 0.001	r = 0.32
	Daily medication	absent: 74% (631)<br>1 - 4 drugs: 25% (211)<br>5 drugs and more: 1.2% (10)<br>Complete: n = 852	absent: 44% (18)<br>1 - 4 drugs: 49% (20)<br>5 drugs and more: 7.3% (3)<br>Complete: n = 41	χ^2	p < 0.001	V = 0.16

Cohort[1]	Variable	DA-negative[2]	DA-positive[2]	Test[3]	pFDR[4]	Effect size[5]
	Dizziness acute COVID-19	29% (247)<br>Complete: n = 852	51% (21)<br>Complete: n = 41	χ^2	p = 0.038	V = 0.1
	Forgetfulness acute COVID-19	32% (270)<br>Complete: n = 852	63% (26)<br>Complete: n = 41	χ^2	p < 0.001	V = 0.14
	Forgetfulness sub-acute COVID-19	24% (207)<br>Complete: n = 852	54% (22)<br>Complete: n = 41	χ^2	p < 0.001	V = 0.14
	Number of acute symptoms	Median = 12 [IQR: 6 - 18]<br>Range: 0 - 39<br>Complete: n = 851	Median = 16 [IQR: 11 - 22]<br>Range: 0 - 34<br>Complete: n = 41	Mann-Whitney	p = 0.018	r = 0.11
	Number of acute neurocognitive symptoms	Median = 0 [IQR: 0 - 2]<br>Range: 0 - 3<br>Complete: n = 852	Median = 2 [IQR: 1 - 3]<br>Range: 0 - 3<br>Complete: n = 41	Mann-Whitney	p = 0.0032	r = 0.12
	Number of sub-acute neurocognitive symptoms	Median = 0 [IQR: 0 - 1]<br>Range: 0 - 3<br>Complete: n = 852	Median = 1 [IQR: 0 - 2]<br>Range: 0 - 3<br>Complete: n = 41	Mann-Whitney	p = 0.0041	r = 0.12

[1]AT: Austria, IT: Italy.

[2]For categorical variables: percentage of the complete answers (n individuals). DA-negative: no pre-existing depression/anxiety, DA-positive: pre-existing depression/anxiety.

[3]Statistical test used for the AT vs IT comparison.

[4]Test p value corrected for multiple comparisons with Benjamini-Hochberg (FDR) method

[5]Effect size: Wilcoxon r or Cramer's V.

Supplementary Figures

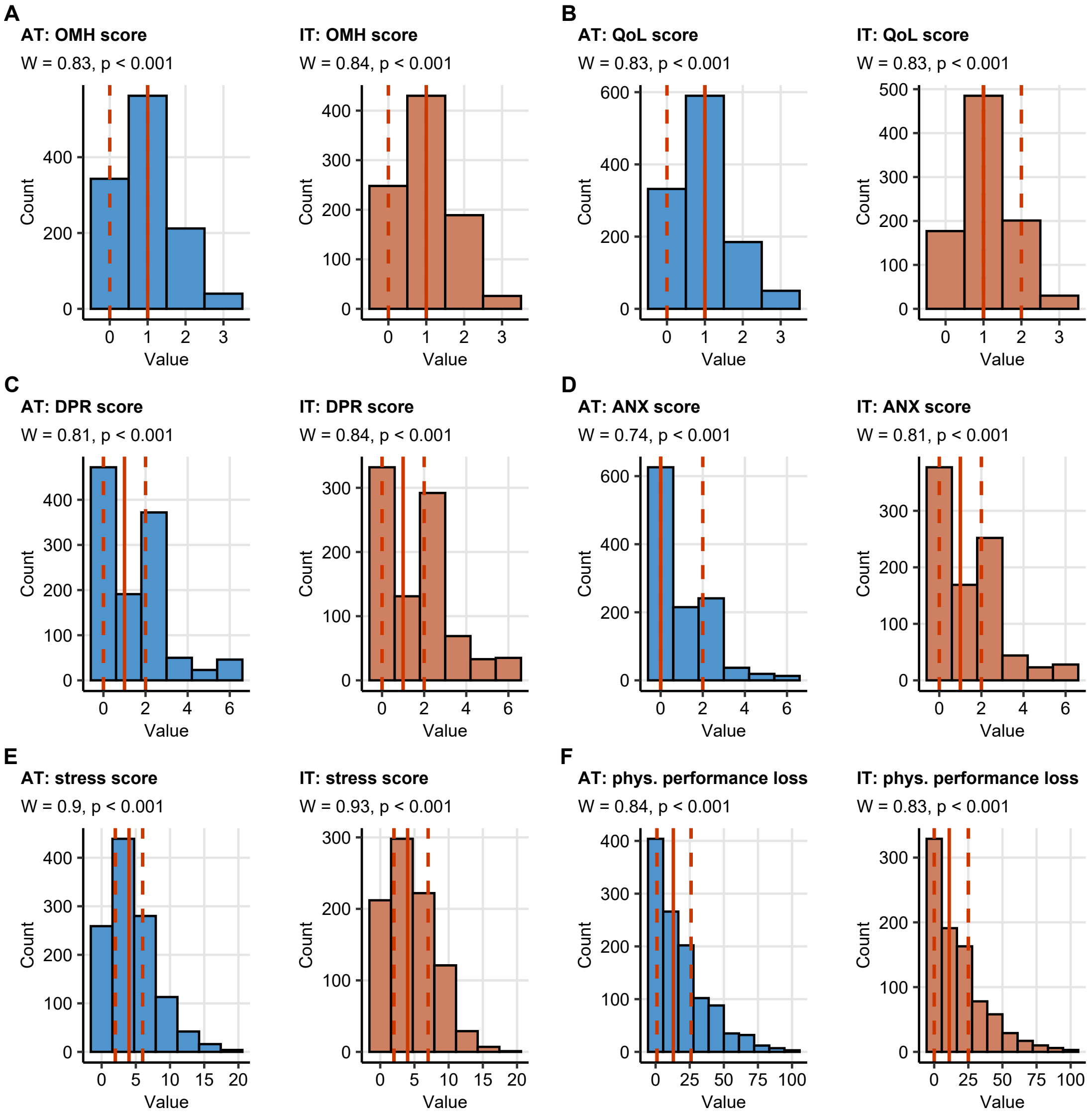

Supplementary Figure S1. Value distribution of the mental health and quality of life scores, stress score and physical performance loss.

Variable distribution was presented as histograms, solid lines represent medians, dashed lines represent 25th and 75th percentile. Normality was assessed by Shapiro-Wilk test. The values of the test statistic and p are presented in the plot headings.

AT: Austria cohort, IT: Italy cohort, OMH: overall mental health score, QoL quality of life score, DPR: depression score, ANX: anxiety score, phys.: physical.

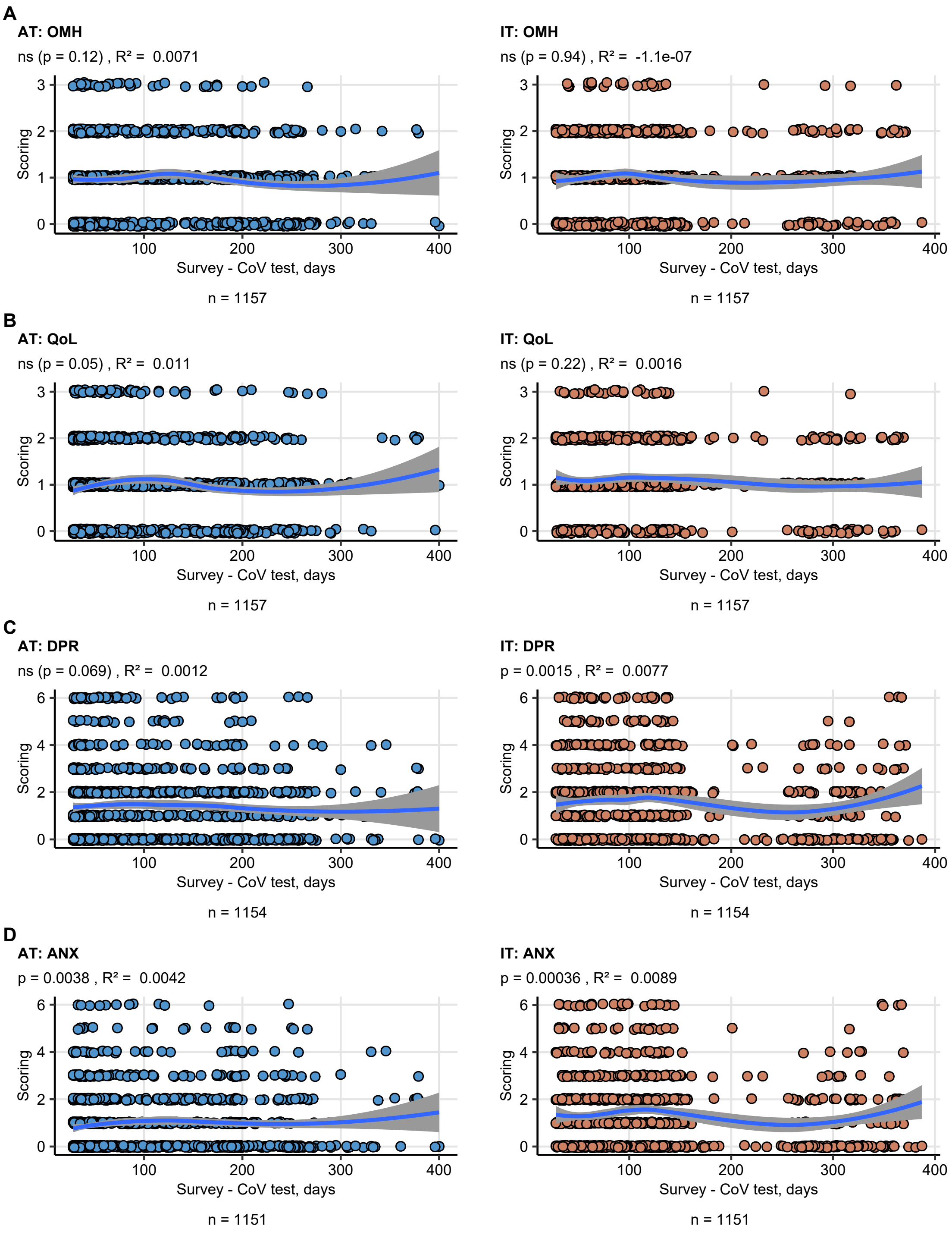

Supplementary Figure S2. Effect of the observation time on mental health and quality of life scoring.

Effects of the survey participation - SARS-CoV-2 diagnosis time interval on the mental health and quality of life scoring was investigated with univariate Poisson general additive modeling (GAM, cubic spline base, k = 20 base dimension). Trend significance corrected for multiple testing with Benjamini-Hochberg method and fraction of the scoring variance explained by the observation time expressed as R^2 are presented in the plot captions. Each point in the plot represents a single observations, blue lines with gray ribbons represent LOESS (locally weighted scatterplot smoothing) trends with 95% confidence regions. Numbers of complete observations are shown under the plots.

AT: Austria cohort, IT: Italy cohort, OMH: overall mental health score, QoL quality of life score, DPR: depression score, ANX: anxiety score, phys.: physical.

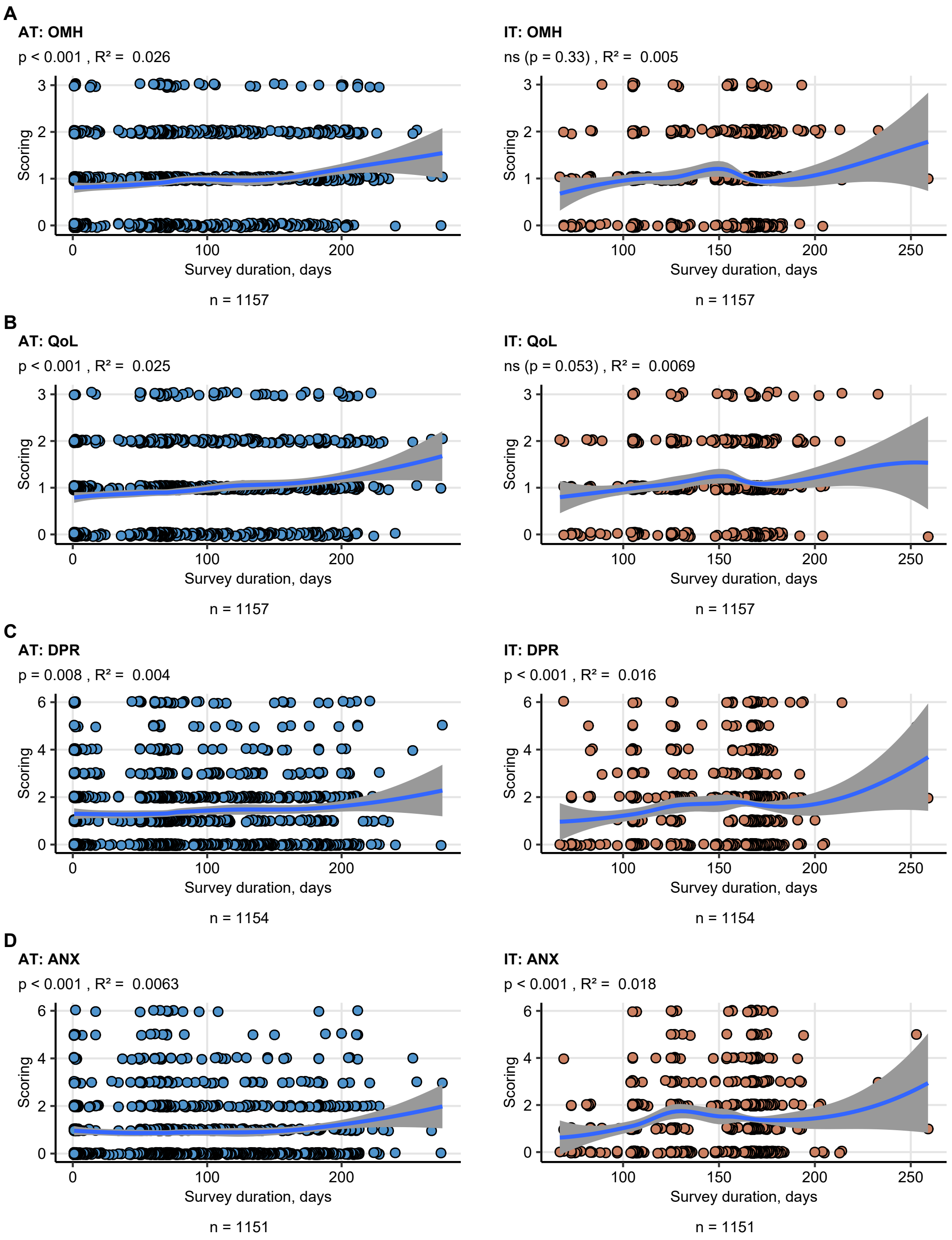

Supplementary Figure S3. Effect of the survey duration on mental health and quality of life scoring.

Effects of the survey duration time on the mental health and quality of life scoring was investigated with univariate Poisson general additive modeling (GAM, cubic spline base, k = 20 base dimension). Trend significance corrected for multiple testing with Benjamini-Hochberg method and fraction of the scoring variance explained by the survey duration expressed as R^2 are presented in the plot captions. Each point in the plot represents a single observations, blue lines with gray ribbons represent LOESS (locally weighted scatterplot smoothing) trends with 95% confidence regions. Numbers of complete observations are shown under the plots.

AT: Austria cohort, IT: Italy cohort, OMH: overall mental health score, QoL quality of life score, DPR: depression score, ANX: anxiety score, phys.: physical.

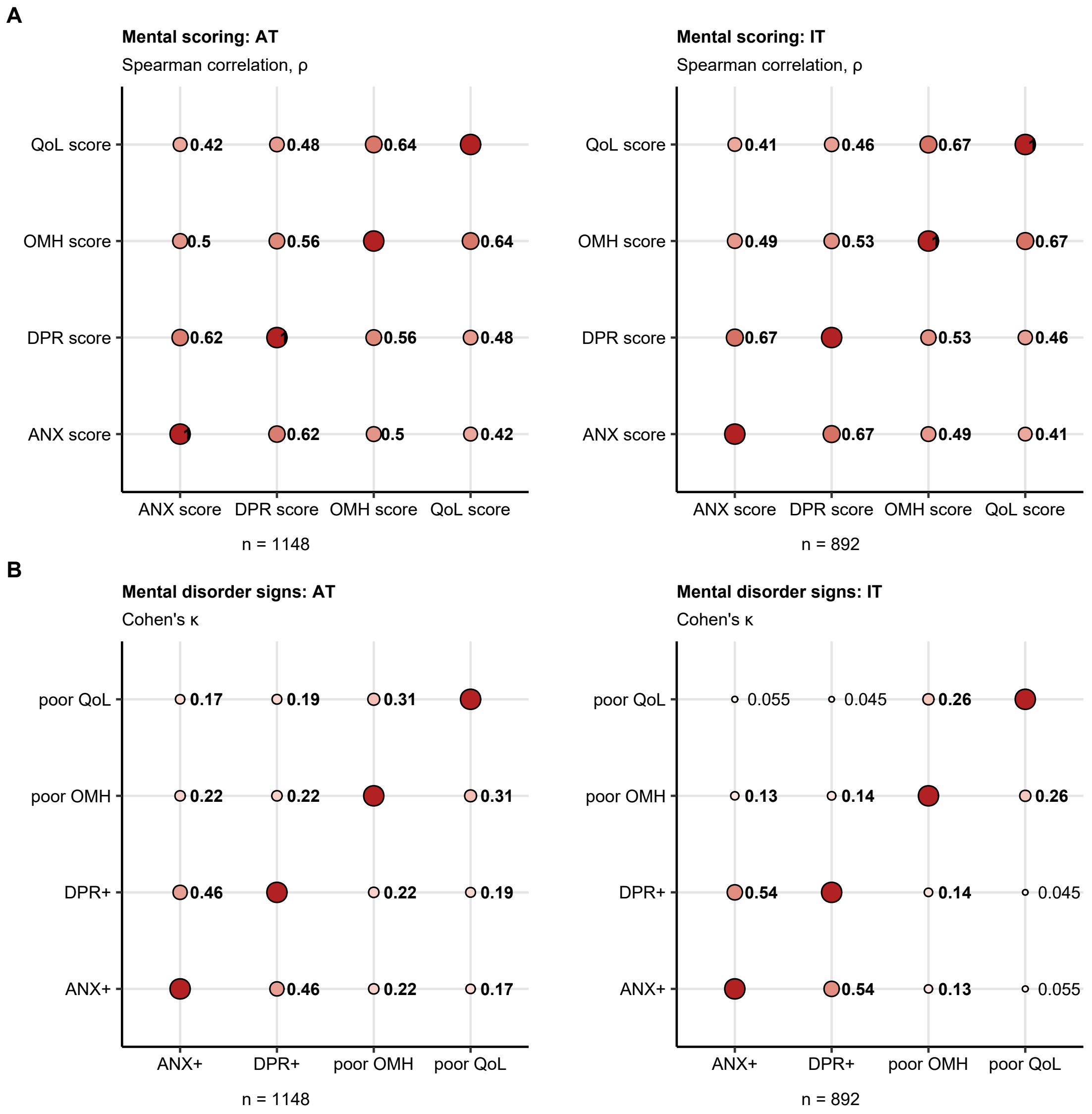

Supplementary Figure S4. Inter-correlation of the mental health and quality of life score variables.

Correlation of the mental health and quality of life scoring (**A**) and overlap between poor self-reported overall mental health, poor quality of life, anxiety and depression sign (**B**) were investigated by Spearman correlation and Cohen's κ, respectively. Values of the correlation/overlap statistics are presented in bubble plots. Point size and color corresponds to the statistic value, the values are indicated next to the plots. Significant ρ and κ statistic values are labeled in bold. Numbers of complete observations are shown under the plots.

AT: Austria cohort, IT: Italy cohort, OMH: overall mental health, QoL quality of life, DPR: depression, ANX: anxiety.

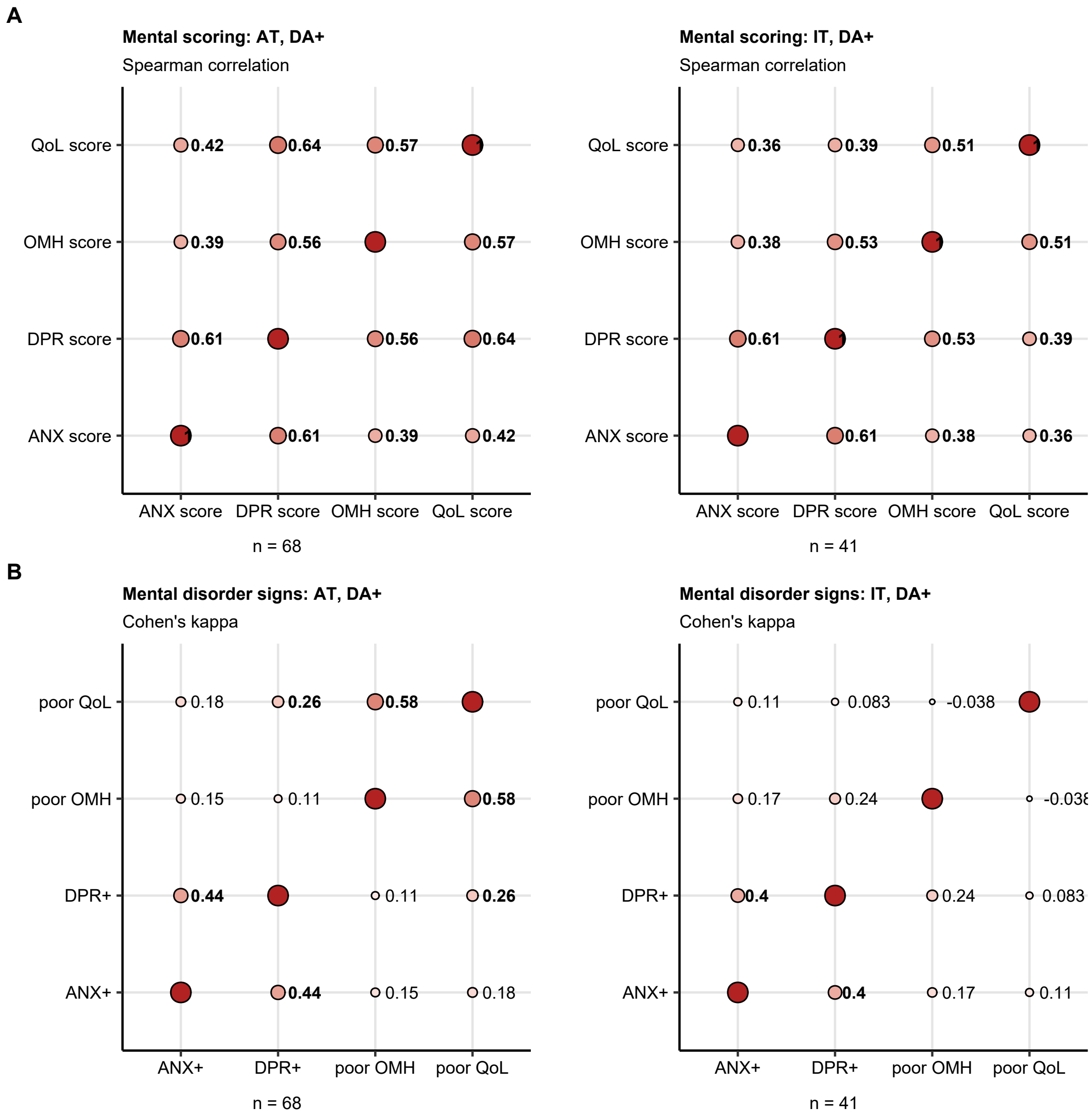

Supplementary Figure S5. Inter-correlation of the mental health and quality of life score variables in participants with pre-existing depression or anxiety.

Correlation of the mental health and quality of life scoring (**A**) and overlap between poor self-reported overall mental health, poor quality of life, anxiety and depression sign (**B**) were investigated in the participants with pre-existing depression/anxiety by Spearman correlation and Cohen's κ, respectively. Values of the correlation/overlap statistics are presented in bubble plots. Point size and color corresponds to the statistic value, the values are indicated next to the plots. Significant ρ and κ statistic values are labeled in bold. Numbers of complete observations are shown under the plots.

AT: Austria cohort, IT: Italy cohort, OMH: overall mental health, QoL quality of life, DPR: depression, ANX: anxiety.

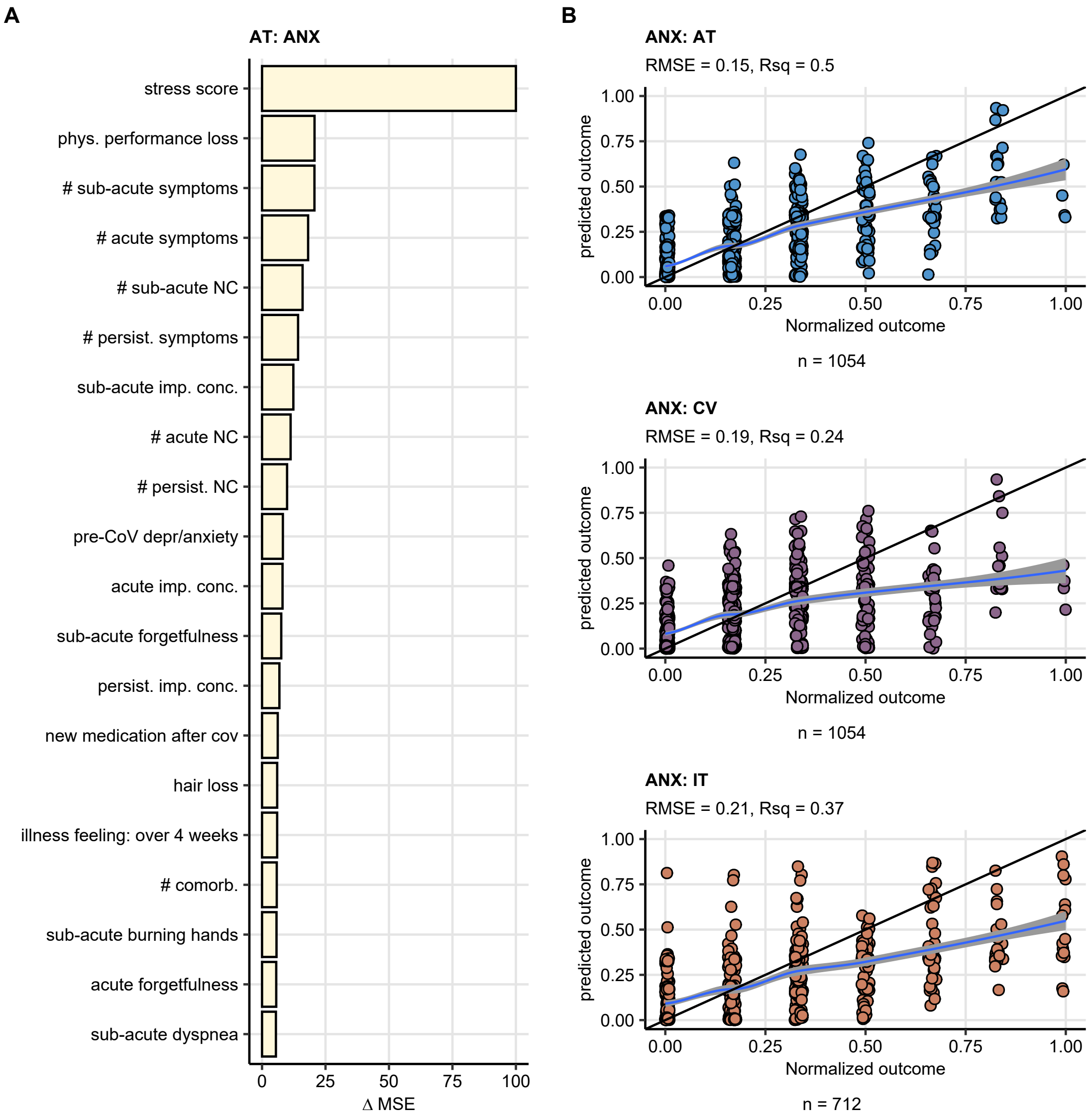

Supplementary Figure S6. Random Forest modeling of the anxiety scoring.

The effects of 201 demographic, clinical, socioeconomic and psychosocial factors (**Supplementary Table S1**) on the anxiety (ANX) scoring were modeled with Random Forest technique. Numeric variables were minimum/maximum normalized prior to modeling. The model was trained in the Austria (AT) cohort, 10-fold cross-validated (CV) and its predictions validated in the Italy (IT) cohort. Explanatory variable importance was estimated in the AT cohort by unbiased ΔMSE statistic. Numbers of complete observations are indicated in (**B**).

(A) Importance statistic values for the top 20 most influential explanatory variables in the AT cohort.

(B) Predicted and actual ANX scoring. Each point represents a single observation, solid black lines represent the optimal calibration line (slope 1, intercept 0). Blue lines with gray regions represent LOESS (locally weighted scatterplot smoothing) trends with 95% confidence intervals. Root mean squared error (RMSE) and R^2 statistic values are presented in the plot captions.

phys.: physical, #: number of, persist.: persistent, imp. conc.: impaired concentration, comorb.: comorbidities, NC: neurocognitive symptoms.

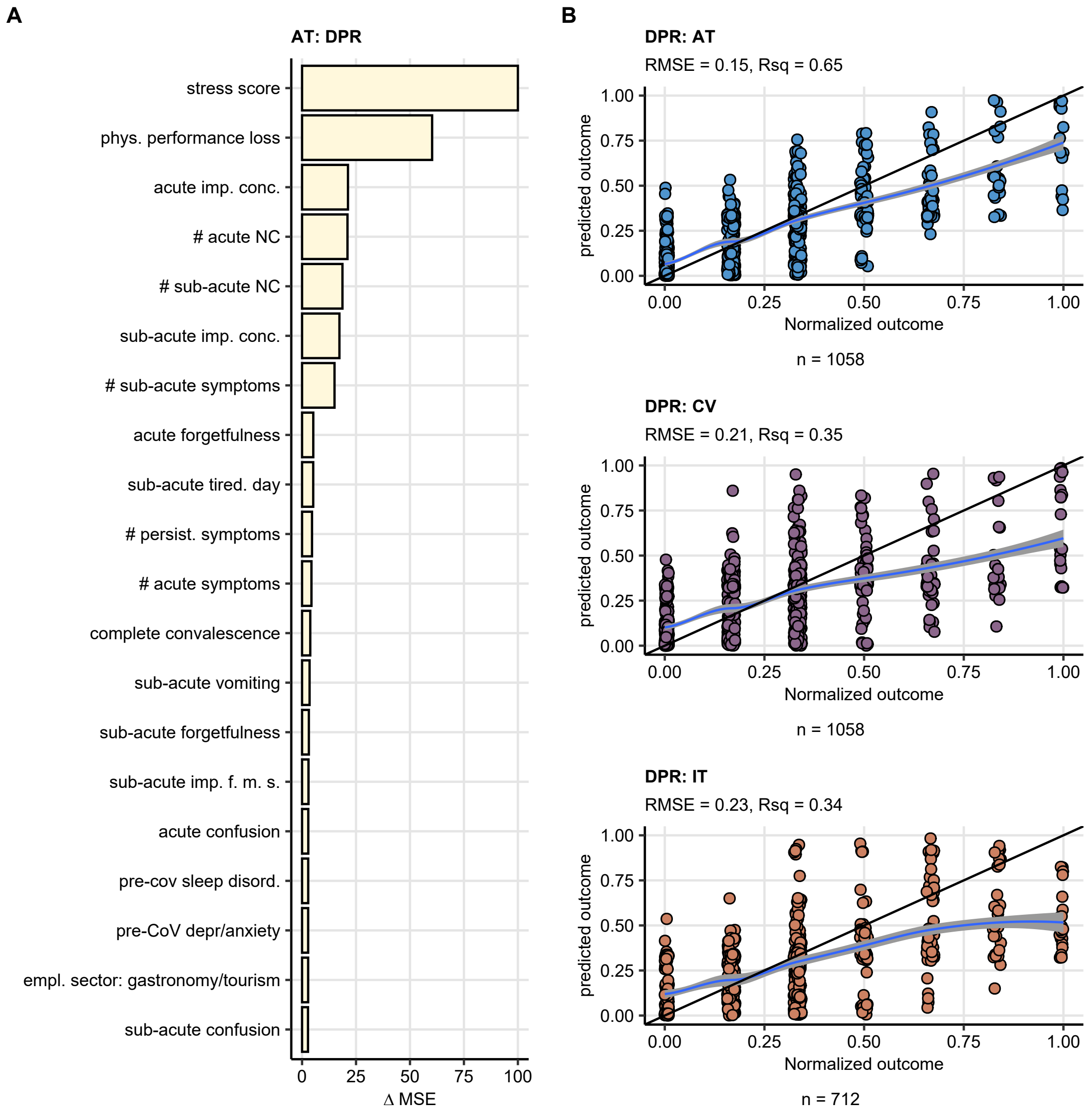

Supplementary Figure S7. Random Forest modeling of the depression scoring.

The effects of 201 demographic, clinical, socioeconomic and psychosocial factors (**Supplementary Table S1**) on the depression (DPR) scoring were modeled with Random Forest technique. Numeric variables were minimum/maximum normalized prior to modeling. The model was trained in the Austria (AT) cohort, 10-fold cross-validated (CV) and its predictions validated in the Italy (IT) cohort. Explanatory variable importance was estimated in the AT cohort by unbiased *Δ*MSE statistic. Numbers of complete observations are indicated in (**B**).

(A) Importance statistic values for the top 20 most influential explanatory variables in the AT cohort.

(B) Predicted and actual ANX scoring. Each point represents a single observation, solid black lines represent the optimal calibration line (slope 1, intercept 0). Blue lines with gray regions represent LOESS (locally weighted scatterplot smoothing) trends with 95% confidence intervals. Root mean squared error (RMSE) and R^2 statistic values are presented in the plot captions.

phys.: physical, #: number of, persist.: persistent, imp. conc.: impaired concentration, tired. day: tiredness at day, imp. f. m. s.: impaired fine motor skills, pre-CoV: before COVID-19, sleep disord.: sleep disorder, depr/anxiety: depression/anxiety, empl. sector: employment sector, NC: neurocognitive symptoms.

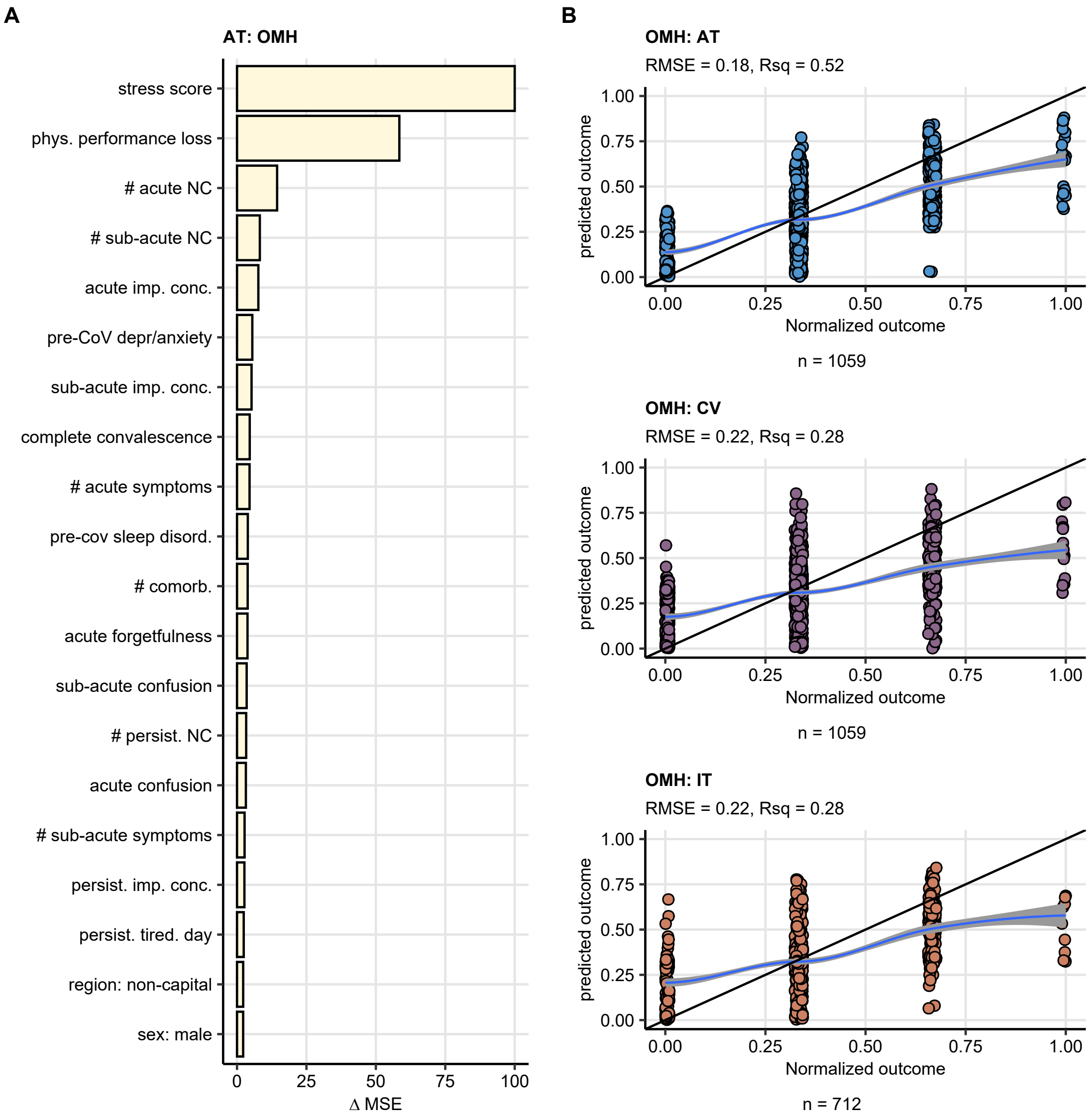

Supplementary Figure S8. Random Forest modeling of the overall mental health scoring.

The effects of 201 demographic, clinical, socioeconomic and psychosocial factors (**Supplementary Table S1**) on the self-perceived overall mental health (OMH) scoring were modeled with Random Forest technique. Numeric variables were minimum/maximum normalized prior to modeling. The model was trained in the Austria (AT) cohort, 10-fold cross-validated (CV) and its predictions validated in the Italy (IT) cohort. Explanatory variable importance was estimated in the AT cohort by unbiased *Δ*MSE statistic. Numbers of complete observations are indicated in (**B**).

(A) Importance statistic values for the top 20 most influential explanatory variables in the AT cohort.

(B) Predicted and actual ANX scoring. Each point represents a single observation, solid black lines represent the optimal calibration line (slope 1, intercept 0). Blue lines with gray regions represent LOESS (locally weighted scatterplot smoothing) trends with 95% confidence intervals. Root mean squared error (RMSE) and R^2 statistic values are presented in the plot captions.

phys.: physical, #: number of, persist.: persistent, imp. conc.: impaired concentration, comorb.: comorbidities, NC: neurocognitive symptoms, pre-CoV: before COVID-19, depr/anxiety: depression/anxiety, sleep disord.: sleep disorder, tired. day: tiredness at day.

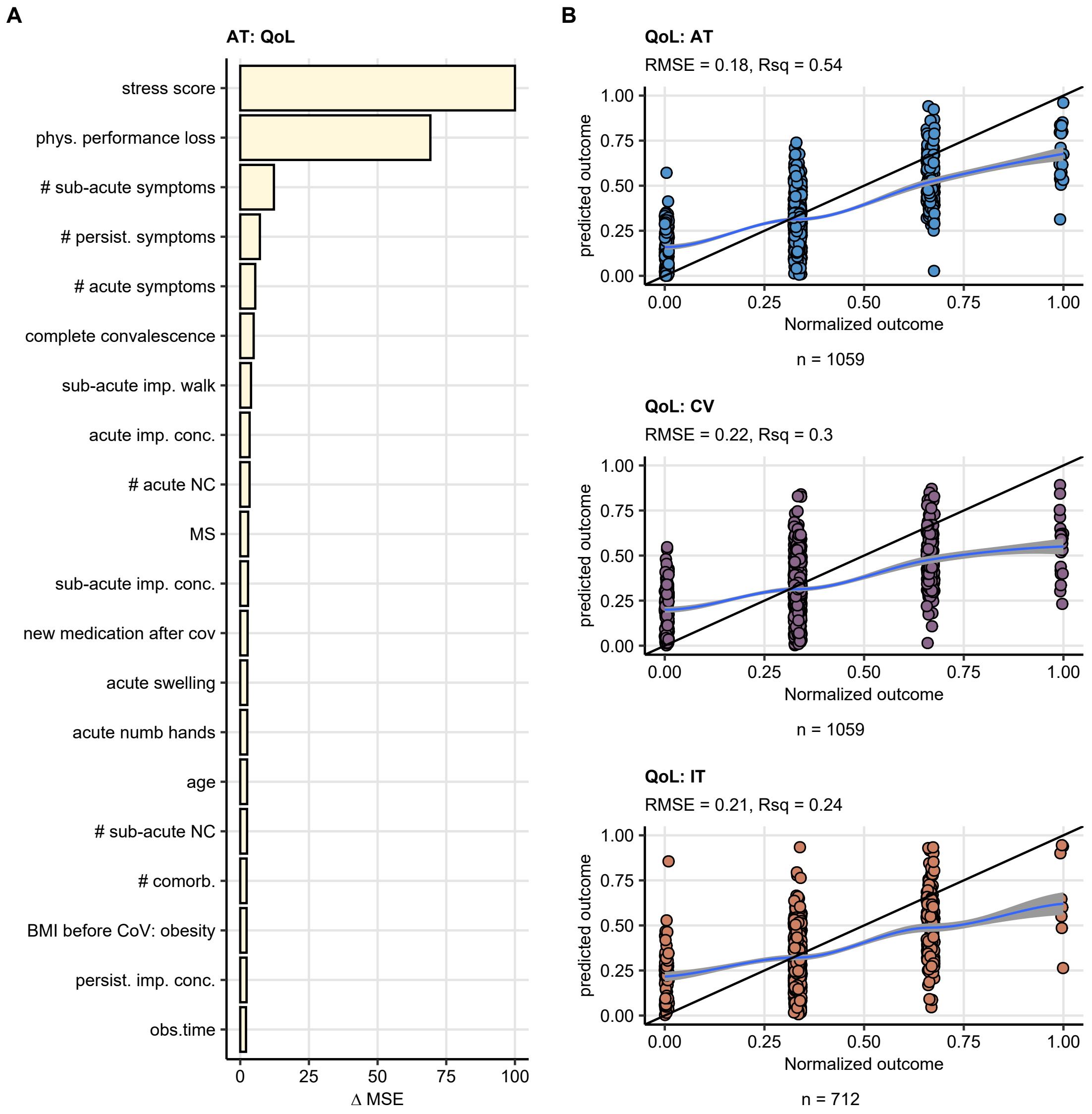

Supplementary Figure S9. Random Forest modeling of the quality of life scoring.

The effects of 201 demographic, clinical, socioeconomic and psychosocial factors (**Supplementary Table S1**) on the self-perceived quality of life (QoL) scoring were modeled with Random Forest technique. Numeric variables were minimum/maximum normalized prior to modeling. The model was trained in the Austria (AT) cohort, 10-fold cross-validated (CV) and its predictions validated in the Italy (IT) cohort. Explanatory variable importance was estimated in the AT cohort by unbiased *Δ*MSE statistic. Numbers of complete observations are indicated in (**B**).

(A) Importance statistic values for the top 20 most influential explanatory variables in the AT cohort.

(B) Predicted and actual ANX scoring. Each point represents a single observation, solid black lines represent the optimal calibration line (slope 1, intercept 0). Blue lines with gray regions represent LOESS (locally weighted scatterplot smoothing) trends with 95% confidence intervals. Root mean squared error (RMSE) and R^2 statistic values are presented in the plot captions.

phys.: physical, #: number of, persist.: persistent, imp. conc.: impaired concentration, comorb.: comorbidities, NC: neurocognitive symptoms, pre-CoV: before COVID-19, depr/anxiety: depression/anxiety, MS: pre-existing multiple sclerosis, BMI: body mass index.

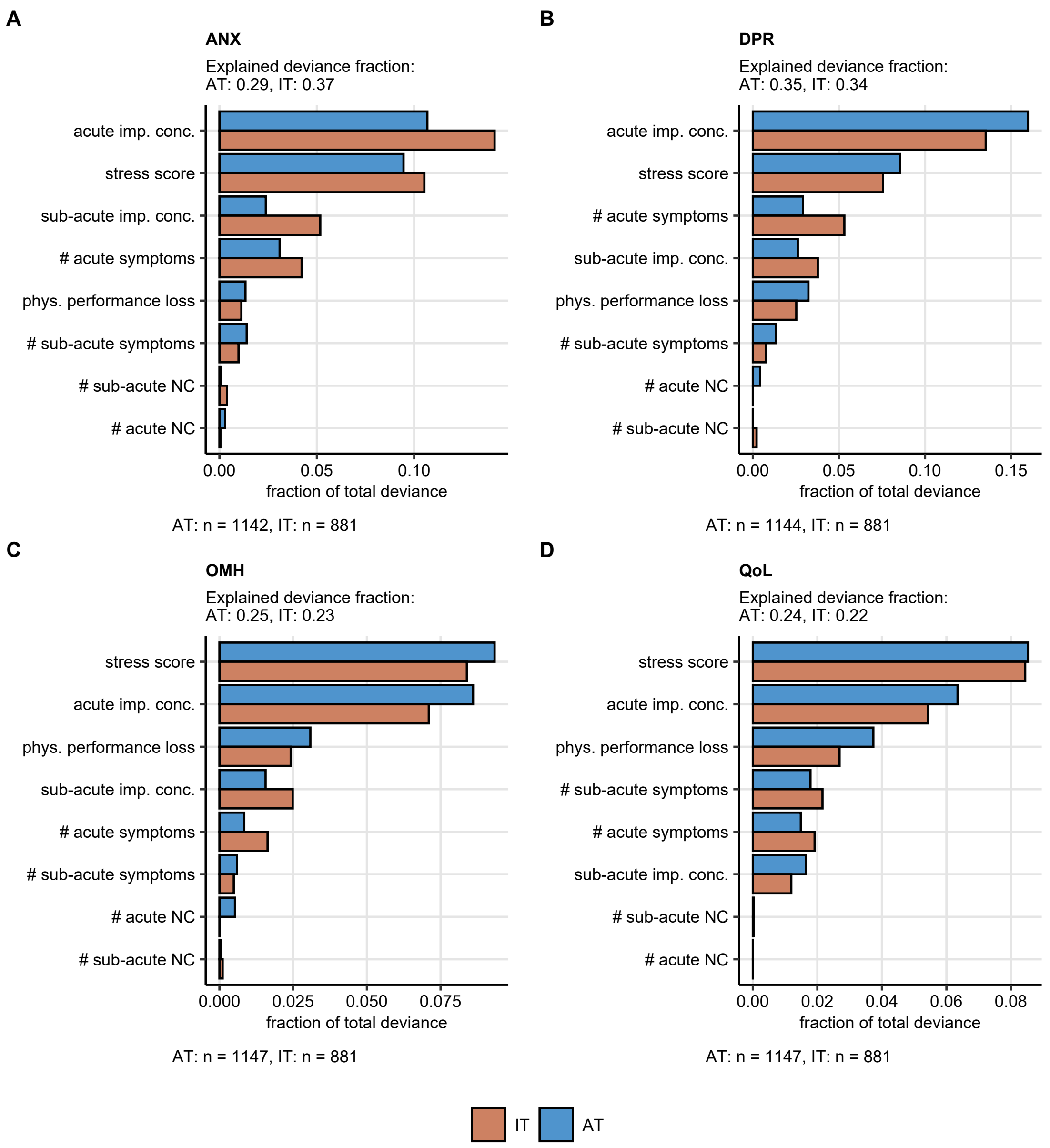

Supplementary Figure S10. Fraction of mental health and quality of life scoring deviance explained by the common most influential factors.

Fraction of deviance explained by the most influential factors for the mental health and quality of life scoring (**Figure 2B**) was investigated by multi-parameter Poisson modeling. Numeric variables were minimum/maximum normalized prior to modeling. The fraction of explained deviance associated with specific model terms was investigated by sequential addition and the values were presented in as bar plots. The total explained deviance

fraction is shown in the plot captions. Numbers of complete observations are indicated under the plots.

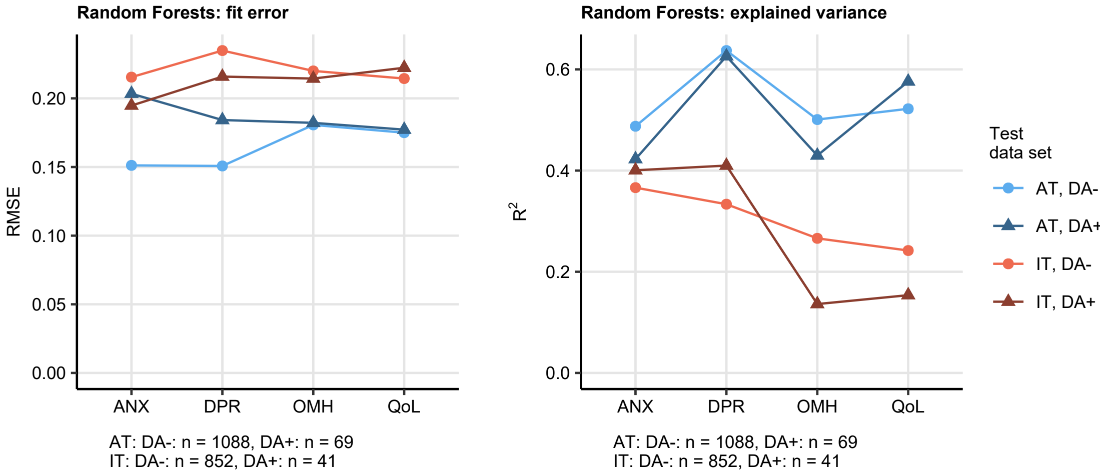

Supplementary Figure S11. Prediction of mental health and quality of life scoring by Random modeling in participants with pre-existing depression or anxiety.

Random Forest models for the mental health and life quality scoring were developed in the Austria cohort as presented in **Figure 2A** and **Supplementary Figures S6 - S9**. Numeric variables were minimum/maximum normalized prior to modeling. The model performance measured by root mean squared error (RMSE) and the fraction of explained variance in mental health and quality of life scoring expressed as R^2 was assessed for model predictions in the subsets of participants with or without depression/anxiety before COVID-19 (DA+ and DA-, respectively) in the Austria (AT) and Italy cohort. Numbers of complete observations are indicated under the plots.

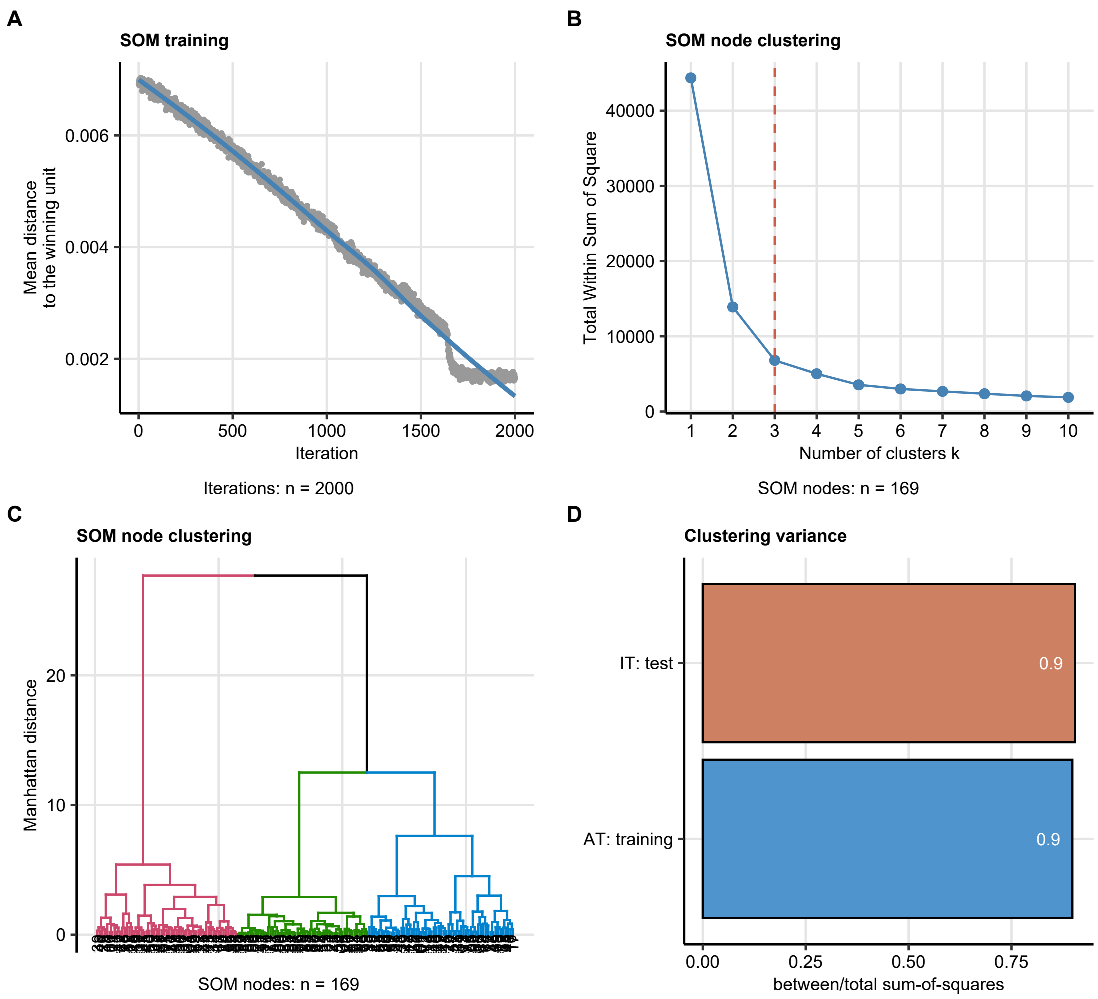

Supplementary Figure S12. Development of the participant clusters in respect to the common most influential factors for mental health and quality of life scoring.

Study participants were assigned to the Low Risk (LR), Intermediate Risk (IR) and High Risk (HR) subsets by clustering in respect to the most influential factors for the mental health and quality of life scoring (**Figure 2B**). Numeric variables were minimum/maximum normalized prior to modeling. The procedure in the training Austria (AT) cohort involved the self-organizing map (SOM, 13 × 13 hexagonal grid, Manhattan distance between participants) and the hierarchical clustering (Ward D2 method, Manhattan distance between the SOM nodes) algorithms. Assignment of the Italy (IT) cohort participants to the clusters was accomplished by the k-nearest neighbors classification.

(A) Training of the SOM grid in the AT cohort. The loss of the mean distance to the winning unit for each SOM node in course of the algorithm iteration (n = 2000 iterations in total) is presented. Points represent single SOM nodel, blue line represents a LOESS trend (locally weighted scatterplot smoothing).

(B) Determination of the optimal number of clusters of the SOM nodes in the AT cohort. Total within-cluster sum-of-squares are presented as a function of the cluster number. The optimal cluster number was determined by the bend of the curve.

(C) Dendrogram of the SOM node clustering. Each leaf represents a single SOM node.

(D) Fraction of clustering variance (between-cluster to total sum-of-squares ratio) in the training AT cohort and the test IT collective.

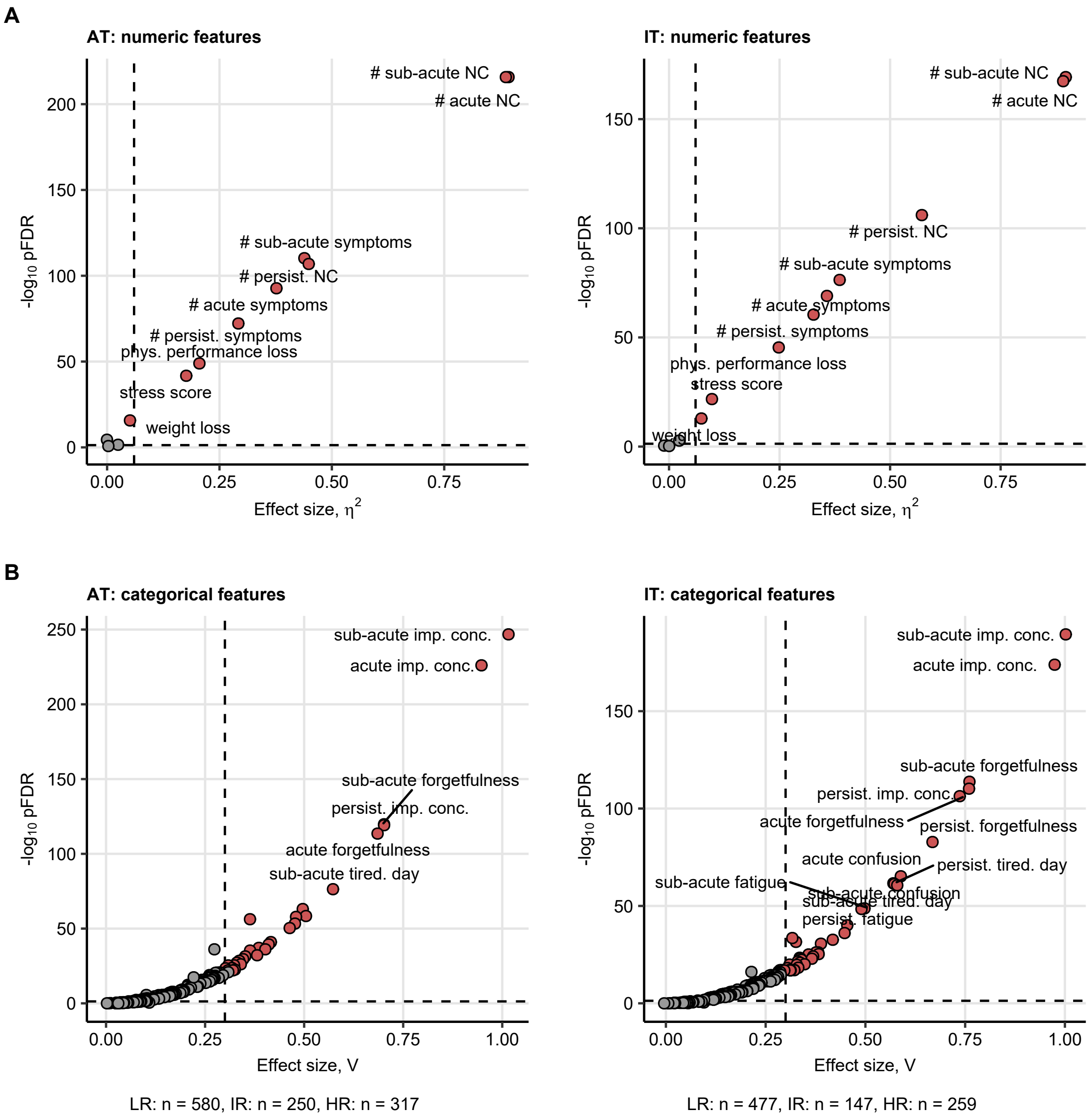

Supplementary Figure S13. Characteristic of the mental disorder risk clusters.

Differences of 201 demographic, clinical, socioeconomic and psychosocial factors (**Supplementary Table S1**) between the mental disorder risk clusters (**Figure 4** and **Supplementary Figure S12**) were investigated by Kruskal-Wallis test and χ^2 test for numeric (**A**) and categorical features (**B**), respectively. Test results were corrected for multiple testing with Benjamini-Hochberg (FDR) method. Effect size was estimated with η^2 statistic for numeric variables and with Cramer's V for categorical variables. Moderately-to-strongly regulated variables were defined by the η^2 > 0.06 or V > 0.30 cutoffs, as appropriate. Variable significance and effect size are presented in the point plots. Dashed lines represent the significance and effect size cutoffs. Strongly regulated variables are

highlighted in red. Numbers of participants assigned to the clusters are presented in (**B**). AT: austria, IT: Italy.

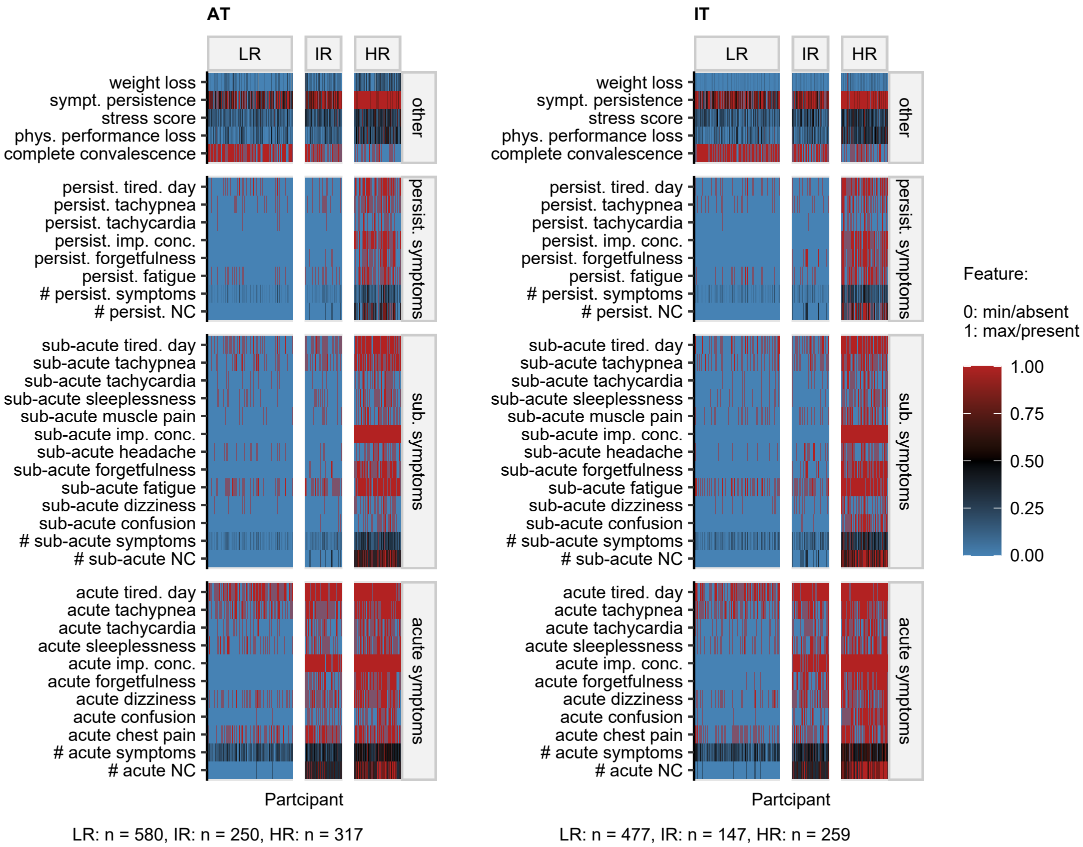

Supplementary Figure S14. Variables significantly differing between the mental disorder risk clusters.

Variables moderately-to-strongly regulated between the mental disorder risk clusters (LR: low risk, IR: intermediate risk, HR: high risk) in the Austria (AT) and Italy (IT) cohort were identified as presented in **Supplementary Figure S13**. Minimum/maximum-normalized values of the variables deemed strongly regulated in both study collectives are presented in heat maps. Numbers of participants assigned to the clusters are presented under the plots.

sympt. persistence: individual time to complete symptom resolution, phys.: physical, persist.: persistent, tired. day: tiredness at day, imp. conc.: impaired concentration, NC: neurocognitive symptoms.

References

1. Sahanic S, Tymoszuk P, Ausserhofer D, Rass V, Pizzini A, Nordmeyer G, Hüfner K, Kurz K, Weber PM, Sonnweber T, et al. Phenotyping of acute and persistent COVID-19 features in the outpatient setting: exploratory analysis of an international cross-sectional online survey. *Clinical Infectious Diseases* (2021) doi: 10.1093/CID/CIAB978

2. Löwe B, Wahl I, Rose M, Spitzer C, Glaesmer H, Wingenfeld K, Schneider A, Brähler E. A 4-item measure of depression and anxiety: Validation and standardization of the Patient Health Questionnaire-4 (PHQ-4) in the general population. *Journal of Affective Disorders* (2010) **122**:86–95. doi: 10.1016/j.jad.2009.06.019

3. Löwe B, Spitzer RL, Zipfel S, Herzog W. Auflage Manual 17.07. (2002). pp.

4. Beutel TF, Zwerenz R, Michal M. Psychosocial stress impairs health behavior in patients with mental disorders. *BMC Psychiatry* (2018) **18**: doi: 10.1186/s12888-018-1956-8

5. Gräfe K, Zipfel S, Herzog W, Löwe B. Screening psychischer störungen mit dem "Gesundheitsfragebogen für Patienten (PHQ-D)". Ergebnisse der Deutschen validierungsstudie. *Diagnostica* (2004) **50**:171–181. doi: 10.1026/0012-1924.50.4.171

6. Health after COVID-19 in Tyrol. Mental Health after COVID-19 in Tyrol. https://im2-ibk.shinyapps.io/mental_health_dashboard/ [Accessed September 9, 2021]

7. Wickham H, Averick M, Bryan J, Chang W, McGowan L, François R, Grolemund G, Hayes A, Henry L, Hester J, et al. Welcome to the Tidyverse. *Journal of Open Source Software* (2019) **4**:1686. doi: 10.21105/joss.01686

8. Wickham H. *ggplot2: Elegant Graphics for Data Analysis*. 1st ed. New York: Springer-Verlag (2016). pp. https://ggplot2.tidyverse.org

9. Wilke CO. *Fundamentals of Data Visualization: A Primer on Making Informative and Compelling Figures*. 1st ed. Sebastopol: O'Reilly Media (2019). pp.

10. Fleiss JL, Cohen J, Everitt BS. Large sample standard errors of kappa and weighted kappa. *Psychological Bulletin* (1969) **72**:323–327. doi: 10.1037/h0028106

11. Benjamini Y, Hochberg Y. Controlling the False Discovery Rate: A Practical and Powerful Approach to Multiple Testing. *Journal of the Royal Statistical Society: Series B (Methodological)* (1995) **57**:289–300. doi: 10.1111/j.2517-6161.1995.tb02031.x

12. Breiman L. Random forests. *Machine Learning* (2001) **45**:5–32. doi: 10.1023/A:1010933404324

13. Kuhn M. Building predictive models in R using the caret package. *Journal of Statistical Software* (2008) **28**:1–26. doi: 10.18637/jss.v028.i05

14. Wright MN, Ziegler A. ranger: A Fast Implementation of Random Forests for High Dimensional Data in C++ and R. *Journal of Statistical Software* (2017) **77**:1–17. doi: 10.18637/JSS.V077.I01

15. Fasiolo M, Wood SN, Zaffran M, Nedellec R, Goude Y. Fast Calibrated Additive Quantile Regression. *https://doiorg/101080/0162145920201725521* (2020) **116**:1402–1412. doi: 10.1080/01621459.2020.1725521

16. Nembrini S, König IR, Wright MN. The revival of the Gini importance? *Bioinformatics* (2018) **34**:3711–3718. doi: 10.1093/BIOINFORMATICS/BTY373

17. Pérez-Silva JG, Araujo-Voces M, Quesada V. nVenn: generalized, quasi-proportional Venn and Euler diagrams. *Bioinformatics* (2018) **34**:2322–2324. doi: 10.1093/BIOINFORMATICS/BTY109

18. Wood SN. Generalized additive models: An introduction with R, second edition. *Generalized Additive Models: An Introduction with R, Second Edition* (2017)1–476. doi: 10.1201/9781315370279/GENERALIZED-ADDITIVE-MODELS-SIMON-WOOD

19. Austrian Agency for Health and Food Safety (AGES). Epidemiologische Abklärung Covid 19. https://www.ages.at/themen/krankheitserreger/coronavirus/epidemiologische-abklaerung-covid-19/ [Accessed August 5, 2021]

20. Istituto Superiore di Sanitá (ISS). Integrated surveillance of COVID-19 in Italy. https://www.epicentro.iss.it/coronavirus/bollettino/Bollettino-sorveglianza-integrata-COVID-19_7-luglio-2021.pdf [Accessed August 5, 2021]

21. Vesanto J, Alhoniemi E. Clustering of the self-organizing map. *IEEE Transactions on Neural Networks* (2000) **11**:586–600. doi: 10.1109/72.846731

22. Kohonen T. *Self-Organizing Maps*. Berlin, Heidelberg: Springer Berlin Heidelberg (1995). pp. doi: 10.1007/978-3-642-97610-0

23. Vesanto J, Vesanto J, Himberg J, Alhoniemi E, Parhankangas J. Self-organizing map in Matlab: the SOM toolbox. *IN PROCEEDINGS OF THE MATLAB DSP CONFERENCE* (1999)35—–40. http://citeseerx.ist.psu.edu/viewdoc/summary?doi=10.1.1.97.179

24. Leng M, Wang J, Cheng J, Zhou H, Chen X. Adaptive semi-supervised clustering algorithm with label propagation. *Journal of Software Engineering* (2014) **8**:14–22. doi: 10.3923/JSE.2014.14.22
